# Hepatocellular carcinoma chemoprevention by targeting the angiotensin-converting enzyme and EGFR transactivation

**DOI:** 10.1172/jci.insight.159254

**Published:** 2022-07-08

**Authors:** Emilie Crouchet, Shen Li, Mozhdeh Sojoodi, Simonetta Bandiera, Naoto Fujiwara, Hussein El Saghire, Shijia Zhu, Tongqi Qian, Fahmida Akter Rasha, Fabio Del Zompo, Stephen C. Barrett, Eugénie Schaeffer, Marine A. Oudot, Clara Ponsolles, Sarah C. Durand, Sarani Ghoshal, Gunisha Arora, Fabio Giannone, Raymond T. Chung, Nevena Slovic, Nicolaas Van Renne, Emanuele Felli, Patrick Pessaux, Joachim Lupberger, Nathalie Pochet, Catherine Schuster, Kenneth K. Tanabe, Yujin Hoshida, Bryan C. Fuchs, Thomas F. Baumert

**Affiliations:** 1 Université de Strasbourg, Inserm, Institut de Recherche sur les Maladies Virales et Hépatiques UMR-S1110, Strasbourg, France.; 2 Division of Gastrointestinal and Oncologic Surgery, Massachusetts General Hospital, Harvard Medical School, Boston, Massachusetts, USA.; 3 Liver Tumor Translational Research Program, Simmons Comprehensive Cancer Center, Division of Digestive and Liver Diseases, Department of Internal Medicine, University of Texas Southwestern Medical Center, Dallas, Texas, USA.; 4 Service de chirurgie viscérale et digestive, Pôle hépato-digestif, Hôpitaux Universitaires de Strasbourg, Strasbourg, France.; 5 Institut hospitalo-universitaire (IHU), Institute for Minimally Invasive Hybrid Image-Guided Surgery, Université de Strasbourg, Strasbourg, France.; 6 Liver Center and Gastrointestinal Division, Massachusetts General Hospital,; 7 Program in Translational NeuroPsychiatric Genomics, Brigham and Women’s Hospital, and Harvard Medical School, Boston, Massachusetts, USA.; 8 Broad Institute of Harvard and Massachusetts Institute of Technology, Cambridge, Massachusetts, USA.; 9 Department of Neurology, Harvard Medical School, Boston, Massachusetts, USA.

**Keywords:** Hepatology, Fibrosis, Liver cancer

## Abstract

Hepatocellular carcinoma (HCC) is a leading cause of death among cirrhotic patients, for which chemopreventive strategies are lacking. Recently, we developed a simple human cell-based system modeling a clinical prognostic liver signature (PLS) predicting liver disease progression and HCC risk. In a previous study, we applied our cell-based system for drug discovery and identified captopril, an approved angiotensin converting enzyme (ACE) inhibitor, as a candidate compound for HCC chemoprevention. Here, we explored ACE as a therapeutic target for HCC chemoprevention. Captopril reduced liver fibrosis and effectively prevented liver disease progression toward HCC development in a diethylnitrosamine (DEN) rat cirrhosis model and a diet-based rat model for nonalcoholic steatohepatitis–induced (NASH-induced) hepatocarcinogenesis. RNA-Seq analysis of cirrhotic rat liver tissues uncovered that captopril suppressed the expression of pathways mediating fibrogenesis, inflammation, and carcinogenesis, including epidermal growth factor receptor (EGFR) signaling. Mechanistic data in liver disease models uncovered a cross-activation of the EGFR pathway by angiotensin. Corroborating the clinical translatability of the approach, captopril significantly reversed the HCC high-risk status of the PLS in liver tissues of patients with advanced fibrosis. Captopril effectively prevents fibrotic liver disease progression toward HCC development in preclinical models and is a generic and safe candidate drug for HCC chemoprevention.

## Graphical abstract

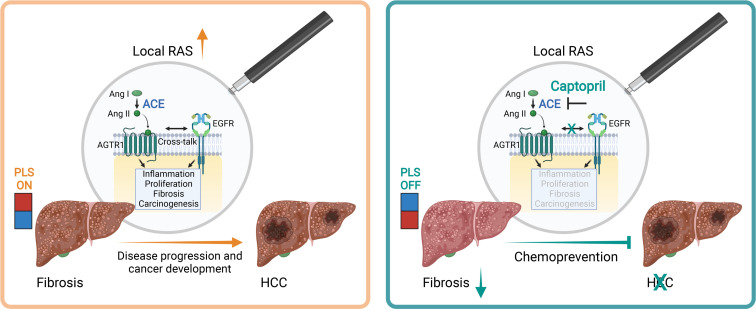

## Introduction

Hepatocellular carcinoma (HCC) is the fourth leading and fastest rising cause of cancer death worldwide and the leading cause of death among cirrhotic patients (1). The main underlying etiologies comprise viral infection (chronic hepatitis B, C, D) or metabolic perturbations such as alcoholic steatohepatitis (ASH), nonalcoholic fatty liver disease (NAFLD), and nonalcoholic steatohepatitis (NASH). Studies in large clinical cohorts have shown that, despite controlling the cause of liver disease such as viral cure, patients with advanced liver fibrosis remain at high risk for HCC (2, 3). While several new modalities for HCC treatment have been approved in the last years (4), there are no approved HCC chemopreventive strategies, despite large research efforts within the last decades (5).

Identification of candidate compounds for HCC chemoprevention has been hampered by the complex cell circuitry driving disease progression and HCC risk and the absence of tractable model systems reflecting human disease. Previously, a pan-etiology 186-gene clinical prognostic liver signature (PLS) predicting liver disease progression, patient survival, and HCC risk was identified and validated in multiple patient cohorts (6–12). We have recently developed a simple human cell-based system modeling the clinical PLS and the major cell circuits driving fibrogenic and carcinogenic disease progression in patients (13). This model, termed cPLS for cell culture PLS, offers opportunities to discover compounds for chemoprevention across the distinct liver cancer etiologies in a fast-track high-throughput screening format using the PLS as readout. In a previous study, we performed an in silico computational screening of more than 20,000 compounds, followed by validation in our cell-based system, and we uncovered captopril as one of the best candidate compounds for HCC chemoprevention (13).

**Figure 1:**
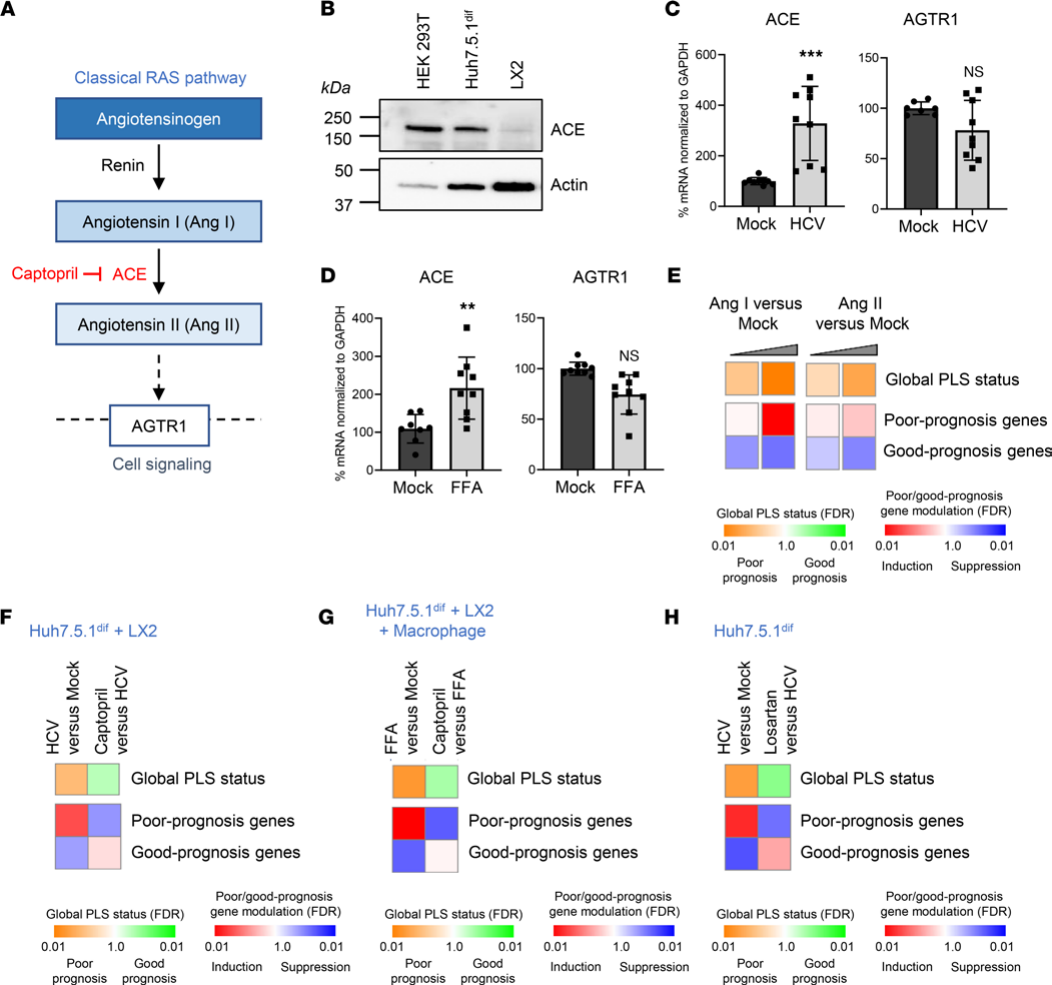
Activation of ACE signaling pathway induces the poor-prognosis PLS associated with HCC risk. (**A**) Simplified schematics of the classical renin-angiotensin system (RAS). Ang I, angiotensin I; Ang II, angiotensin II; ACE, angiotensin converting enzyme; AGTR1, Ang II type 1 receptor. (**B**) ACE is expressed in Huh7.5.1^dif^. HEK 293T, positive control; LX2, hepatic stellate cell line. (**C** and **D**) Expression of ACE and AGTR1 in persistently HCV-infected cells (**C**) or free fatty acid–treated (FFA-treated) cells (**D**) measured by qPCR. Results are from 3 experiments performed in triplicate, *n* = 9 (% mean ± SD; **P < 0.01; ***P < 0.001, unpaired *t* test). (**E**) Ang I and Ang II are mediators of the PLS in the cell-based system. The 32 gene PLS reversal was determined by gene set enrichment analysis (GSEA) using “Mock” nontreated cells as reference. Simplified heatmaps show (top) the classification of PLS status as poor (orange) or good (green) prognosis; (bottom) the significance of induction (red) or suppression (blue) of poor- or good-prognosis genes. One representative experiment out of 2 is shown. (**F**–**H**) Captopril and losartan reverse the poor-prognosis PLS in different cell-based systems. The poor-prognosis PLS was induced in Huh7.5.1^dif^ cells alone or cocultured with LX2 and THP1-derived macrophages by HCV infection or FFA exposure. Cells were treated with captopril 5 μM or losartan 10 μM before PLS assessment. Simplified heatmaps show: (top) the classification of PLS status as poor (orange) or good (green) prognosis; (bottom) the significance of induction (red) or suppression (blue) of poor- or good-prognosis genes. One representative experiment out of 2 is shown. For PLS experiments, normalized enrichment scores (NES) and exact FDR are provided in Supplemental Table 2. See complete unedited blots in the supplemental material.

Captopril is an angiotensin (Ang) converting enzyme (ACE) inhibitor that is primarily used to treat hypertension. ACE is a component of the renin-Ang system (RAS), a key regulator of cardiovascular function and blood pressure. In the classical pathway, the RAS precursor angiotensinogen is produced by the liver and cleaved by an enzyme, called renin, into Ang I. Ang I is then converted into Ang II by ACE. Ang II is the primary effector of the pathway, and it regulates various cell processed such as vasoconstriction, cell proliferation, and inflammation through interaction with the Ang II type 1 receptor (AGTR1) (Figure 1A) (14). In parallel, the alternative RAS pathway fine tunes the effect of the classical RAS pathway through the production of Ang (1–7) by ACE2 (15). The RAS was classically described as a circulating hormonal system. However, the concept of “local” RAS was more recently introduced based on the discoveries of RAS components in different organs and of noncardivoascular effects of the RAS (16). While studies have suggested a functional role for the RAS in liver biology (16), the role of ACE in HCC chemoprevention remains unclear.

Here, we aimed to explore ACE as a therapeutic target for HCC chemoprevention by applying state-of-the-art animal models and perturbation studies in patient-derived models, combined with transcriptomics and proteomics. Moreover, to investigate the clinical translatability of the approach, we studied the impact of captopril on the liver cell circuits predicting fibrosis progression to HCC in cirrhotic patients.

## Results

### Activation of ACE signaling pathway induces the poor-prognosis PLS associated with HCC risk.

To decipher the effect of ACE modulation on the cell circuits driving liver disease progression and HCC risk, we first applied our previously established human liver cell culture cPLS systems (13). These cell-based systems are based on the use of DMSO-differentiated Huh7.5.1 cells (Huh7.5.1^dif^ cells) alone, in coculture with LX2 stellate cells or in triculture with LX2 and THP1-derived macrophages. As a model of chronic liver injury, cells were exposed to persistent hepatitis C virus (HCV) infection or metabolic injury (free fatty acids [FFA] exposure) (13). As a readout, we used a reduced version of the PLS comprising 32 genes bioinformatically selected and validated in several patient cohorts predicting HCC risk and patient outcome (for a PLS gene list, refer to Supplemental Table 1; supplemental material available online with this article; https://doi.org/10.1172/jci.insight.159254DS1) (9, 10, 17).

First, we confirmed that ACE, the captopril target, is expressed in the cPLS models, indicating that the cPLS systems can be used to monitor ACE pathway dysregulation (Figure 1, A–D; for all the full-length immunoblots, please refer to online supplemental materials). Moreover, we observed that ACE expression, but not AGTR1 expression, is increased upon both viral and metabolic injuries, suggesting a link between ACE expression and disease progression (Figure 1, C and D). Dose-dependent induction of the PLS poor-prognosis status by Ang I and Ang II demonstrates the functional activity of ACE and AGTR1 in the cPLS model, as well as the impact of the pathway activation in the modulation of the PLS associated with HCC risk (Figure 1E and Supplemental Table 2). Finally, we observed that captopril and losartan, an inhibitor of AGTR1, reverses the cPLS (Figure 1, F–H, and Supplemental Table 2), confirming that the Ang/ACE/AGTR1 pathway is a mediator of the poor-prognosis PLS associated with poor survival and high HCC risk in patients (6–12).

### Captopril efficiently and safely prevents HCC development in 2 animal models for advanced fibrotic liver disease.

We then sought to validate the therapeutic and chemopreventive effect of captopril in a rat model diethylnitrosamine (DEN) injection. The DEN rat model is considered as one of the best rodent models recapitulating the serial development of fibrosis, cirrhosis, and HCC formation (10). The DEN model was also chosen because it most closely mimics global liver transcriptome dysregulation in human cirrhosis with striking PLS induction (10, 18). At the very onset of fibrosis at 8 weeks, captopril was administered via oral gavage. All animals were sacrificed at 18 weeks.

**Figure 2:**
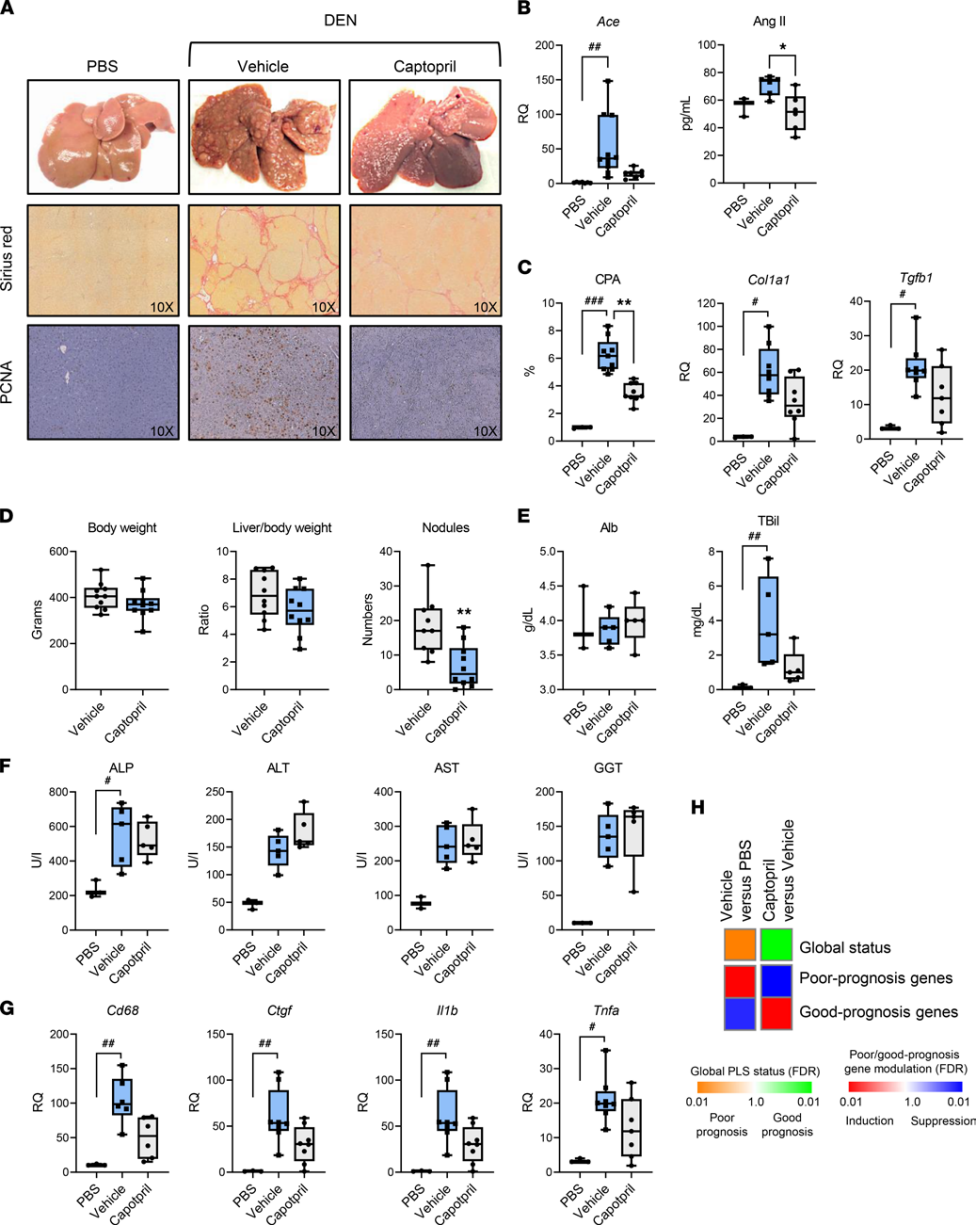
In vivo validation of captopril for HCC chemoprevention in a DEN fibrosis/HCC rat model. (**A**–**D**) Captopril alleviates fibrosis progression and prevents HCC development in vivo. Male Wistar DEN-treated rats received vehicle control or captopril for 10 weeks (vehicle, *n* = 10; captopril, *n* = 10). (**A**) Representative morphometric analysis of liver slices at the time of sacrifice are shown. Picrosirius red staining was used to quantify collagen cross bridging. Proliferating cell nuclear antigen (PCNA) staining was used to quantify cell proliferation. Original magnification, ×100. (**B**) Assessment of the RAS component in vivo. ACE expression was assessed by qPCR and Ang II serum levels by ELISA. (**C**) Collagen proportional area (CPA) expressed in percentage of liver tissue and measurement of the fibrosis markers by qPCR. (**D**) The body weight, the liver/body weight ratio, and the number of total tumors was plotted for each animal. (**E** and **F**) Measurement of albumin and total bilirubin, serum transaminases (alanine aminotransferase [ALT], aspartate aminotransferase, [AST], alkaline phosphatase [ALP], and γ-glutamyl transferase [γGT]) are shown. (**G**) Captopril decreases liver inflammation in vivo. Measurement by qPCR of the macrophage marker *Cd68* and proinflammatory/fibrotic cytokines. For **B**–**G**, boxes represent the 75th and 25th percentiles, the whiskers represent the most extreme data points, and the horizontal bar represents the median. ^#^*P* < 0.05, ^##^*P* < 0.01, and ^###^*P* < 0.001, vehicle vs. PBS. *P < 0.05, captopril vs. vehicle. Kruskal-Wallis test followed by Dunn’s multiple comparisons test was used to compare the 3 groups (**B**, **C**, **E**–**G**); unpaired *t* test was used to compare 2 groups (**D**). (**H**) Captopril reverses the PLS in vivo. PLS induction was determined by GSEA using PBS animals as reference. Simplified heatmaps show PLS global status and PLS poor- and good-prognosis gene expression. RQ, relative quantification.

First, we investigated ACE expression and serum levels of Ang II (ACE product) in animals. ACE expression and Ang II levels were increased in DEN-injured rats, unraveling a role of ACE and Ang II as a mediator and therapeutic candidate target in liver disease progression and hepatocarcinogenesis (Figure 2, A and B). Reduced Ang II levels after captopril treatment confirmed target engagement in vivo (Figure 2B).

Next, we studied the functional effect of ACE inhibition on liver disease and HCC. Treatment with captopril markedly reduced liver fibrosis. Captopril treatment reduced the collagen proportional area (CPA) by around 44% compared with DEN injured rats (*P* < 0.001) (Figure 2A, 2C). The expression of the profibrosis markers (*Col1a1* and *Tgfb1*) was also reduced in comparison with DEN injured rats (*P* < 0.05) (Figure 2C). Captopril treatment also had a marked and significant effect on carcinogenesis, as shown by the decrease of gross tumor nodules by 60% (*P* < 0.01) (Figure 2, A and D) and by the decrease of proliferating cell nuclear antigen (PCNA) staining used as a marker for cell proliferation (Figure 2, A and D). Importantly, captopril did not result in detectable liver toxicity, as shown by the measurement of liver function tests (alanine aminotransferase [ALT], aspartate aminotransferase [AST], alkaline phosphatase (ALP), and γ-glutamyl transferase [γGT]) and total bilirubin level (T-Bil) (Figure 2, E and F).

The cirrhotic background also generates a proinflammatory milieu, which can serve to promote carcinogenesis. Captopril treatment decreases expression of proinflammatory and profibrotic markers, including connective tissue growth factor (*Ctgf*), TNF-α (*Tnf-a*), and IL-1β (*Il1b*) (*P* < 0.05) (Figure 2G). The decrease of *Cd68* expression after captopril treatment reflects a reduced macrophage number in the liver, which correlates with the decrease of liver inflammation (Figure 2G). Finally, captopril efficiently and significantly reverted the poor-prognosis status of the PLS in vivo, supporting a chemopreventive effect (Figure 2H). Together, these data demonstrate that captopril effectively and safely prevents fibrotic liver disease progression toward HCC development.

**Figure 3:**
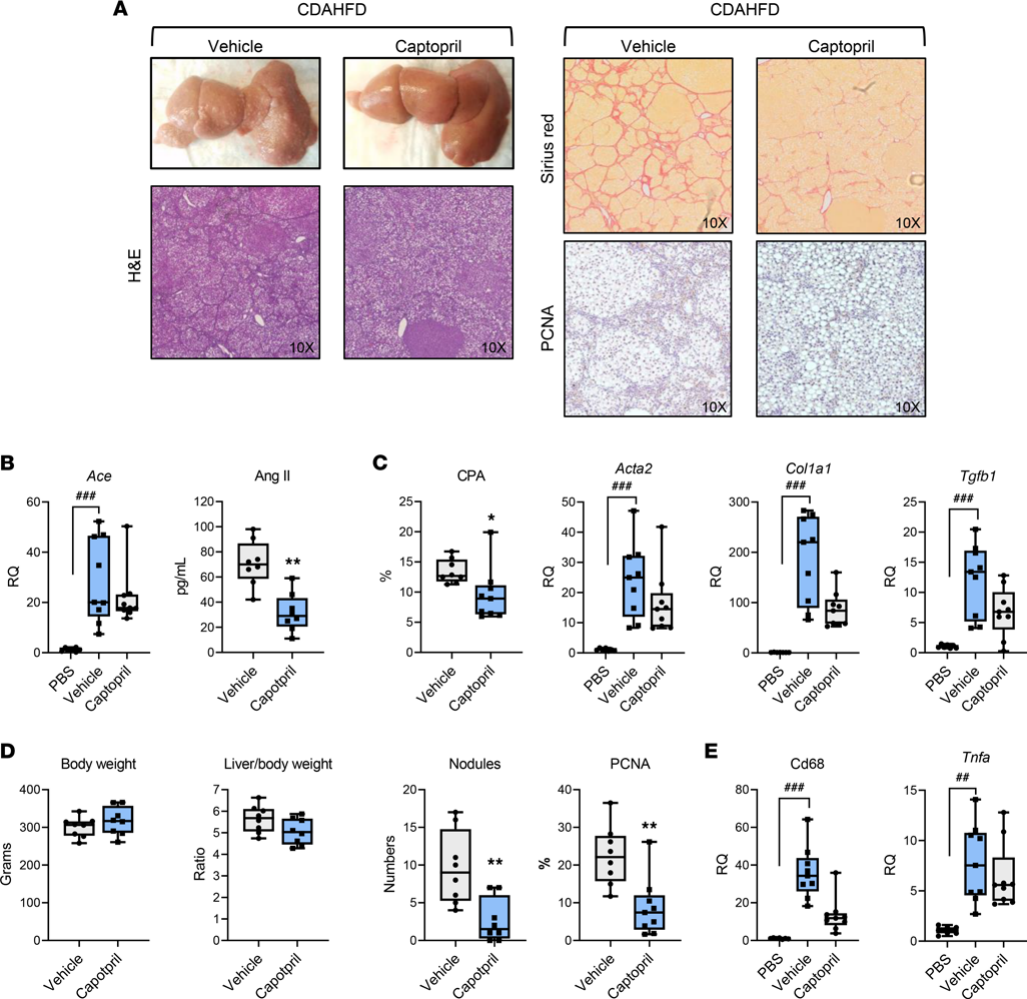
Captopril prevents liver fibrosis progression and cancer development in NASH/HCC rat model. Captopril alleviates fibrosis progression and prevents HCC development in a NASH/HCC rat model. Male Wistar rats were subjected to either standard chow or choline-deficient, L-amino acid–defined, high-fat diet (CDAHFD) for a total of 18 weeks. Oral gavage of captopril was initiated after 6 weeks of CDAHFD diet following the onset of fibrosis (vehicle, *n* = 8; captopril, *n* = 8). (**A**) Representative morphometric analysis of liver slices at the time of sacrifice are shown. Picrosirius red staining was used to quantify collagen cross bridging. Proliferating cell nuclear antigen (PCNA) staining was used to quantify cell proliferation. Original magnification, ×10. (**B**) Assessment of the RAS component in vivo. Angiotensin II (Ang II) serum levels were assessed by ELISA, and ACE expression was assessed by qPCR. (**C**) Collagen proportional area (CPA) expressed in percentage of liver tissue and measurement of the fibrosis markers *Acta2*, *Col1a1*, and *Tgfb1* by qPCR. (**D**) The body weight, the liver/body weight ratio, and the number of total tumors and PCNA quantification was plotted for each animal. (**E**) Captopril decreases liver inflammation in vivo. Measurement of the tumor the macrophage marker *Cd68* and of *Tnfa* by qPCR is shown. For **B**–**E**, boxes represent the 75th and 25th percentiles, the whiskers represent the most extreme data points, and the horizontal bar represents the median. ^##^*P* < 0.01, ^###^*P* < 0.001, vehicle vs. PBS. *P < 0.05, **P < 0.01, captopril vs. vehicle. Kruskal-Wallis test followed by Dunn’s multiple comparisons test was used to compare the 3 groups (**B**, **C**, and **E**); unpaired *t* test was used to compare 2 groups (**D**). RQ, Relative Quantification.

To validate the key findings in a second and complementary model, we investigated the HCC chemopreventive effect of captopril in a potentially novel, diet-only rat model of HCC induced by choline-deficient, L-amino acid–defined, high-fat diet (CDAHFD) (19) (Figure 3). This diet result in a progressive liver pathology, with development of steatosis, inflammation, dysregulation of metabolism, and fibrosis, which characterize human NASH. Similar to our results in the DEN rat model, captopril markedly and significantly inhibited fibrosis and hepatocarcinogenesis (Figure 3).

**Figure 4:**
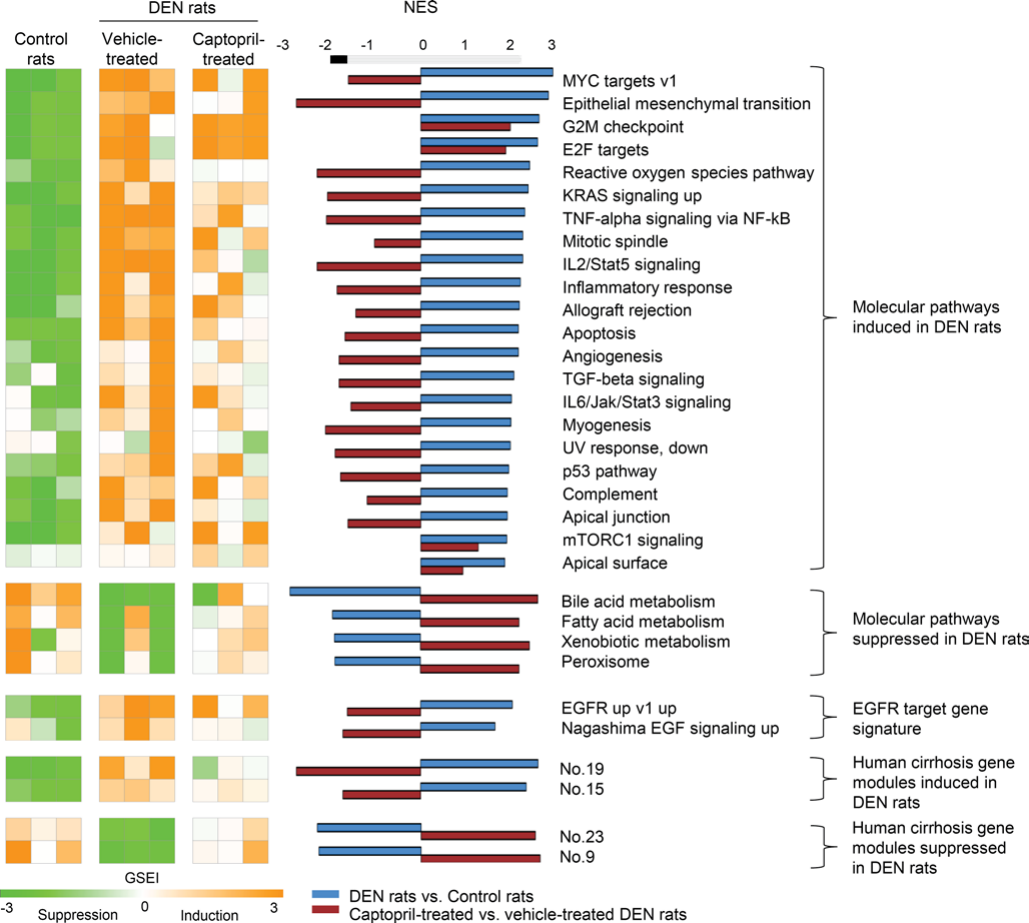
Dysregulated modular pathways in the DEN rats and their modulation by captopril. Heatmap shows induction (orange) or suppression (green) of the molecular pathways and human cirrhosis gene modules (10) in the DEN-treated rats (middle 3 columns) compared with the control rats (left 3 columns), as well as how the pathways are modulated by captopril treatment (right 3 columns) as gene set enrichment index (GSEI) calculated from GSEA. The normalized enrichment score (NES) is shown in the middle panel as magnitude and direction of the molecular pathway modulation in the comparison between the DEN rats and the control rats (blue bars; positive NES indicates induction in the DEN rats compared with the control rats) and the comparison between the captopril- versus vehicle-treated DEN rats. Three animal per groups were analyzed. Molecular pathways dysregulated in comparison between the DEN rats and the control rats were selected at a significance cutoff of FDR < 0.005. DEN, diethylnitrosamine.

To understand the functional impact of captopril on liver disease biology and HCC development in the context of advanced fibrosis, we next performed RNA-Seq analysis on rat liver tissues (Figure 4). Captopril suppressed the expression of several key pathways mediating fibrogenesis and inflammation, such as TGF-β and TNF-α/NF-κB signaling, as well as pathways involved in carcinogenesis, such as cMyc, KRas, and IL-6/STAT3 signaling (20, 21). In addition, captopril improved gene expression of the key liver metabolic pathways (i.e., bile acid and fatty acid metabolisms) (Figure 4).

To investigate the clinical translatability of the approach, we studied the impact of captopril on the liver cell circuits predicting fibrosis progression to HCC in cirrhotic patients. In our previous study, a transcriptome meta-analysis of human cirrhotic tissues identified global regulatory gene networks in cirrhotic liver driving disease progression and HCC risk (10). Interestingly, we demonstrated that the low-dose DEN–induced HCC rat model shows comparable induction of these cirrhosis gene modules (10). Therefore, the reversal of the dysregulated cirrhosis gene modules in DEN-injected animals, as well as the PLS, can be monitored to assess the efficacy of antifibrotic and HCC chemopreventive strategies. We then assessed the effects of captopril treatment on the human gene modules. We observed that captopril treatment restored expression of the gene modules 23 and 9, which are impaired in cirrhotic tissues and are associated with normal hepatocyte metabolism (e.g., lipid and glucose metabolism, coagulation, wound healing), suggesting an improvement of liver function (Figure 4). Moreover, captopril suppresses the gene modules 19 and 15 associated with extracellular matrix remodeling, the profibrogenic CTGF signaling, and cell cycle check point, indicating a decrease in profibrogenic and procarcinogenic signals (Figure 4). Finally, we observed that captopril strongly suppresses the EGFR signaling pathways, a well-described driver of liver disease (18).

Together, these results demonstrate the antifibrogenic and chemopreventive effects of captopril and corroborate the clinical translatability of our chemopreventive strategy.

### Mechanistic studies uncover crosstalk of the Ang/AGTR1 and EGFR signaling in HCC chemoprevention.

**Figure 5:**
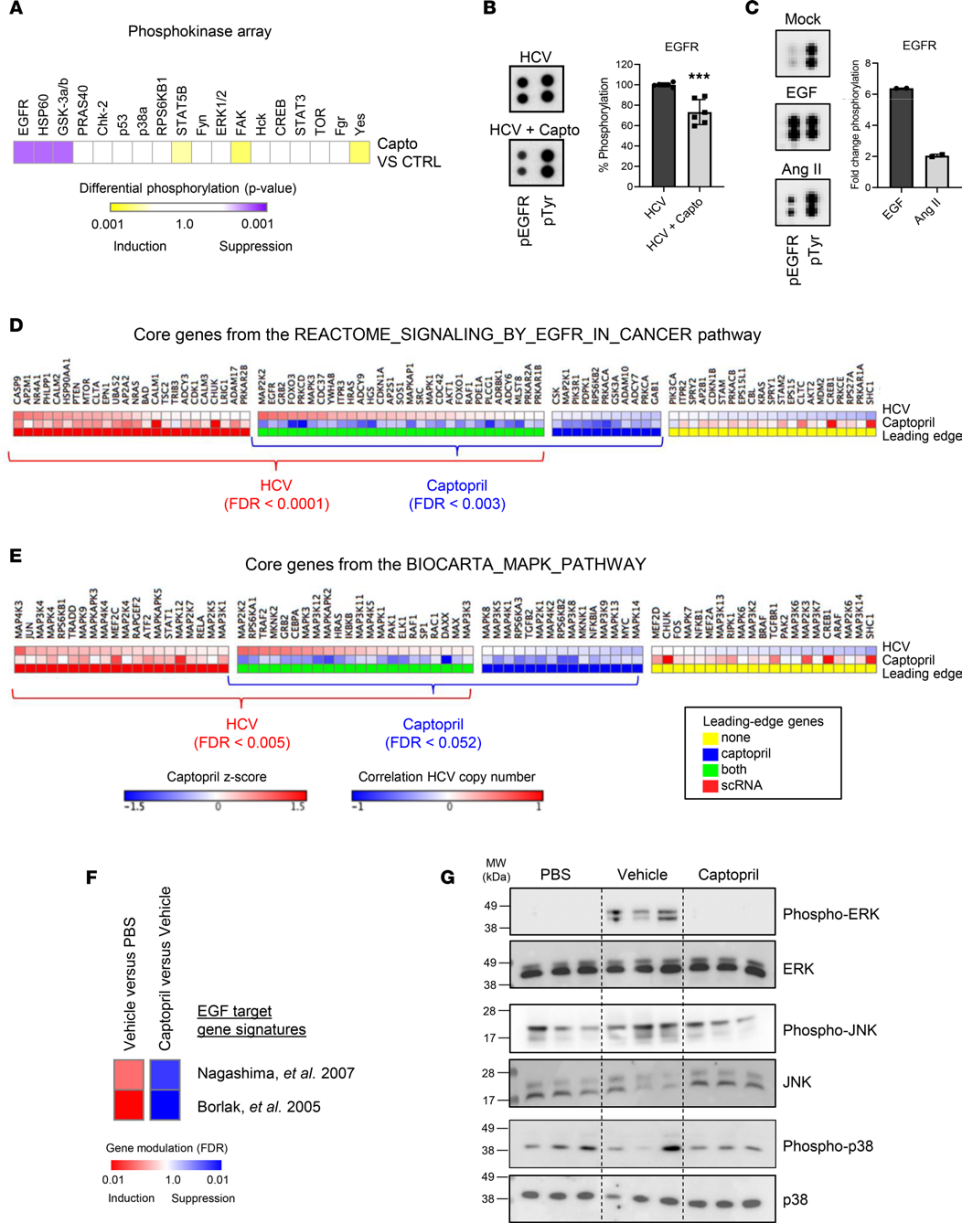
Captopril prevents HCC development by targeting the angiotensin-induced EGFR transactivation. (**A**) Effect of captopril on receptor tyrosine kinase (RTK) phosphorylation in the cell-based model. Heatmap shows the significance of induction (yellow) or suppression (purple) of protein phosphorylation in drug-treated samples compared with untreated controls. Results show means from 3 independent experiments performed in duplicate. (**B**) Captopril treatment decreases EGFR phosphorylation in HCV-infected cells. Panphosphorylation of EGFR was assessed using phosphoarray. Results are shown as mean ± SEM of integrated dot blot densities from 3 independent experiments performed in duplicate (*n* = 6). ***P < 0.001, unpaired *t* test. (**C**) EGFR is a downstream effector of Ang II. Panphosphorylation of EGFR was assessed using phosphoarray in Huh7.5.1^dif^ cells. The graph shows the quantification of dot blot intensities (fold change of phosphorylated EGFR). One representative experiment out of 2 is shown. (**D** and **E**) Captopril treatment significantly repressed EGFR and downstream MAPK pathway genes induced in response to HCV infection at the single-cell level. Heatmaps show core EGFR signaling genes that are modulated by captopril treatment (blue) or HCV infection (red), both (green) or none (yellow), as defined by “leading-edge” genes driving the enrichment score in GSEAs. The effect of captopril on these genes is indicated by *Z* scores (middle row), while the effect of HCV infection is indicated by the Pearson correlation of the expression levels with the HCV viral load (top row). Significance of gene expression modulation was determined using the hypergeometric test. (**F** and **G**) EGFR activation is suppressed by captopril treatment in a rat model for fibrosis and HCC. (**F**) Captopril reverses 2 EGF target gene signature in vivo (GSEA). (**G**). Western blot analysis showing Erk1/2, JNK, and p38 expression and phosphorylation in the liver of animals (3 animals per group). See complete unedited blots in the supplemental material.

To go deeper in the mechanism of action of ACE inhibition and HCC prevention, we investigated the liver disease signaling pathways affected by ACE inhibition using phospho-kinase array analyses of the HCV cPLS system. We observed that captopril significantly modulated the phosphorylation of different kinases playing a functional role in cell metabolism, inflammation, and immune responses (Figure 5A). Interestingly, captopril suppressed EGFR activation, as observed in the RNA-Seq analyses (Figure 4 and Figure 5, A and B). Activation of EGFR by Ang II stimulation of the Huh7.5.1^dif^ cells confirmed the crosstalk between Ang and the EGFR pathways (Figure 5C). Furthermore, single-cell RNA-Seq (scRNA-Seq) analyses in the cell-based system confirmed that captopril treatment significantly repressed EGFR and the downstream MAPK pathway genes that are induced in response to HCV infection (Figure 5, D and E). Together, these results indicate a crosstalk between the RAS and the EGFR pathway upon liver injury.

We next assessed the contribution of the EGFR pathway in the induction of the liver cell circuits associated with poor survival and high HCC risk. Transcriptome-based network analysis in multietiology clinical patient cohorts (HCV-, HBV-, and alcohol-related liver diseases) identified 2 major gene networks, in which epidermal growth factor (EGF) or p53/Myc plays a central regulatory role (Supplemental Figure 1). Activation of the EGF receptor (EGFR)/MAPK pathway was also observed in the cPLS liver disease model in an etiology-independent manner, as shown by enhanced EGFR phosphorylation (Supplemental Figure 2A), upregulation of *EGF/EGFR* expression (Supplemental Figure 2B), and induction of experimentally defined EGF target gene signatures (22, 23) (Supplemental Figure 2C). Induction of the EGFR/MAPK pathway was correlated with the magnitude of the poor-prognosis PLS induction at the single-cell level (Supplemental Figure 2D). Moreover, cell stimulation by EGF was sufficient to induce the poor-prognosis pattern of the PLS (Supplemental Figure 2E) and pharmacological inhibition of the pathway by erlotinib (EGFR inhibitor), tipifarnib (Ras inhibitor), and Fr180204 (Erk1/2 inhibitor) reversed the PLS induction in a varying degree (Supplemental Figure 2E). These findings demonstrate that the EGFR/MAPK pathway is a key mediator of the clinical PLS prognosis status.

Interestingly, we observed a reversion of the Ang II–induced poor-prognosis PLS by erlotinib, highlighting the key role of the EGFR pathway in the Ang II–induced HCC high-risk signature (Supplemental Figure 2F). Given these results in cell-based models, we hypothesized that inhibition of the Ang/EGFR axis is most likely responsible for the inhibition of fibrotic liver disease progression toward HCC development in vivo. We, therefore, investigated whether captopril treatment inhibits the EGFR pathway in the DEN rat model for progressive liver disease and HCC. Transcriptome profiling of livers from captopril-treated rats showed a suppression of EGFR target gene signatures (Figure 5F). Moreover, Western blot analyses show a decrease in activation of the downstream MAPK pathway (p-p38, pERK1/2, and p-JNK), corroborating the mechanistic data obtained in cell culture (Figure 5G). Collectively, these results suggest that captopril prevents fibrotic liver disease progression toward HCC development by targeting the Ang-EGFR crosstalk in vivo.

### scRNA-Seq from patient liver tissues uncovers that liver RAS activation results from crosstalk between hepatocyte and the liver microenvironment.

**Figure 6:**
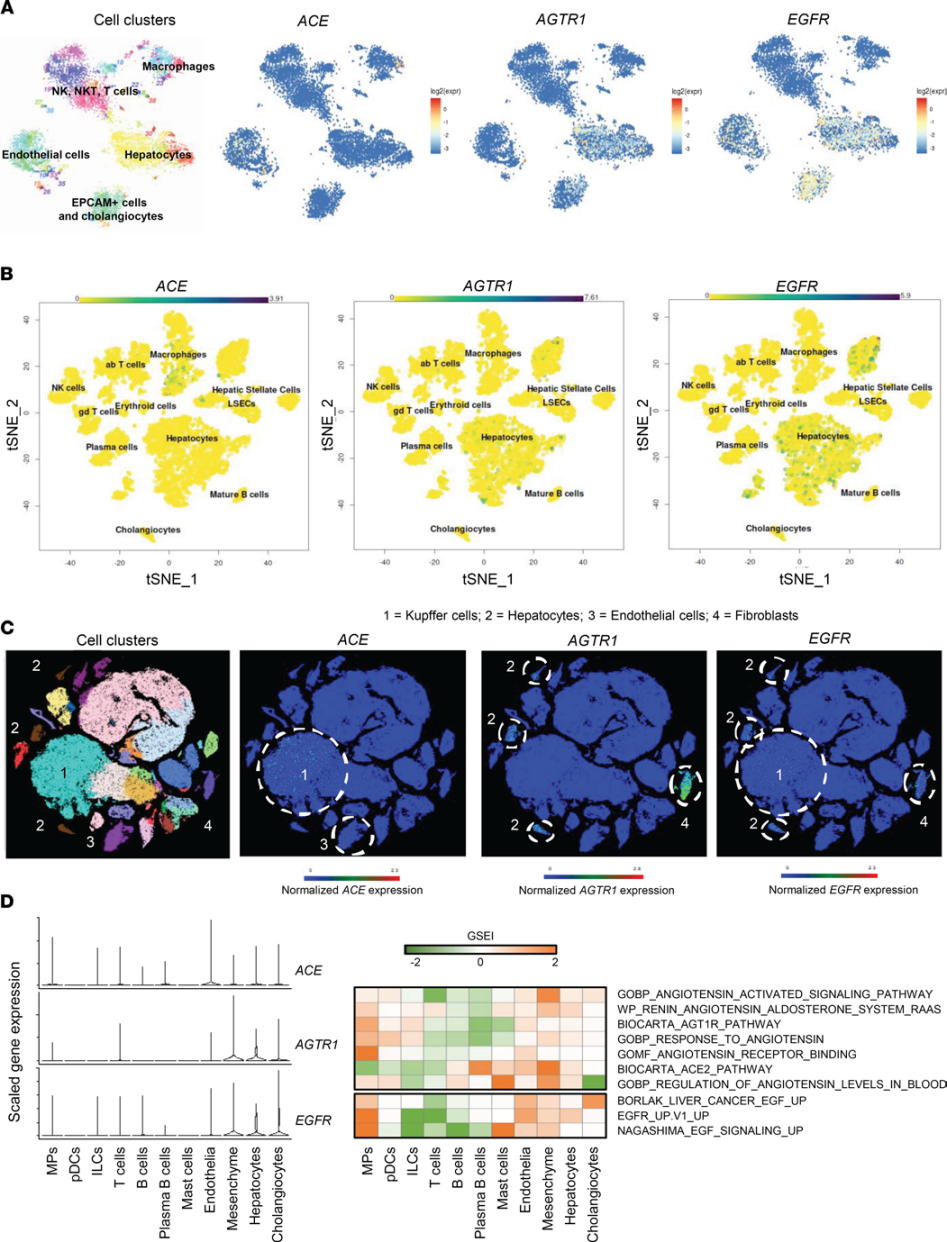
Single-cell RNA-Seq from patient liver tissues uncovers that liver RAS activation results from a crosstalk between hepatocyte and the liver microenvironment. (**A**–**C**) t-SNE map of single-cell transcriptomes from normal liver tissue of donors without history of chronic liver disease highlighting the main liver cell compartments and expression t-SNE map of *ACE*, *AGTR1*, and *EGFR*. Data extracted from **A** (27), **B** (26), and **C** (25). Cells sharing similar transcriptome profiles are grouped by clusters and each dot represents 1 cell. The color bar indicates log2 normalized expression. (**D**) Scaled gene expression of *ACE*, *AGTR1*, and *EGFR* and RAS-related signature enrichment (gene set enrichment index [GSEI]) across the main cell compartments from cirrhotic patient liver tissues. Data extracted from ref. 28.

The crosstalk between hepatocytes and the surrounding microenvironment plays an important role in liver disease progression and hepatocarcinogenesis (24). To obtain insights in the potential role of the microenvironment in the liver RAS, we analyzed expression of the RAS pathway components in recently published human liver cell atlases (25–28). In healthy liver tissue (25–27), epithelial cells, including hepatocytes and cholangiocytes, and fibroblasts show highest expression of the Ang II receptor AGTR1 (Figure 6, A–C). ACE is expressed with the highest level in macrophages and endothelial cells (Figure 6, A–C). In contrast, in patient cirrhotic liver tissues (28), ACE is detected in epithelial cells, including hepatocytes and cholangiocytes, with an enrichment of RAS-related signatures (gene set enrichment index [GSEI]) in these cell compartments (Figure 6D), suggesting an increase in RAS signaling in diseased tissues. Of note, ACE expression in macrophages was confirmed at the RNA and protein levels in THP1-derived macrophages, with the highest expression in M2 macrophages harboring an immunosuppressive phenotype and associated with cancer development (Supplemental Figure 3), supporting an involvement of ACE pathways in carcinogenesis (26, 29). scRNA-Seq expression profiles of the different RAS component also suggest that activation of the local RAS pathway in the liver may be based on a crosstalk between hepatocytes and nonparenchymal cells. Interestingly, EGFR is coexpressed with AGTR1 in hepatocytes and fibroblasts, corroborating our mechanistic data demonstrating the Ang-EGFR crosstalk (Figure 6D).

### Validation of captopril as a target for HCC chemoprevention in patient-derived liver tissues and disease models.

**Figure 7:**
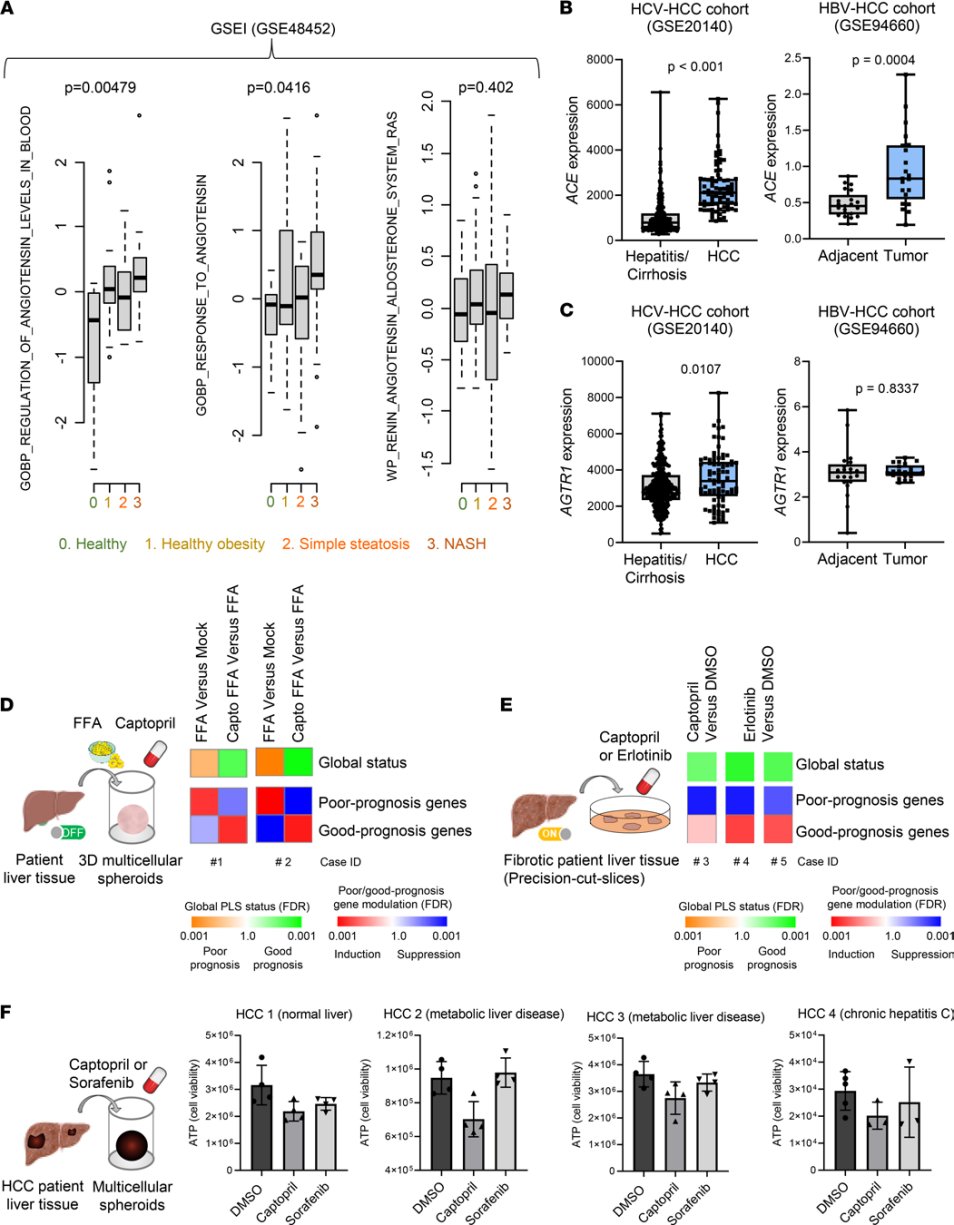
Validation of the potential therapeutic effect of captopril in patient-derived liver tissues. (**A**) Enrichment of RAS-related signatures in NAFLD/NASH cohorts (GSEI). Jonckheere-Terpstra test was used to test gradual increase of continuous values along with ordinal variables (NASH stage). Exact *P* values are shown. GSE48452: healthy obesity, *n* = 27; NASH, *n* = 18. (**B** and **C**) *ACE* and *AGTR1* expression in liver tissues of clinical cohorts with chronic liver disease and HCC. GSE20140: hepatitis/cirrhosis, *n* = 307; HCC, *n* = 80. Exact *P* values are shown (Mann-Whitney *U* test). GSE94660: paired samples, *n* = 21. Exact *P* values are shown (paired *t* test). (**D**) Captopril reverts the FFA-induced poor-prognosis PLS in culture of patient-derived multicellular spheroids (2 patients without history of chronic liver disease). PLS induction was determined by GSEA using “Mock” nontreated spheroids as reference, and PLS reversion was determined by comparing FFA + captopril–treated spheroids to FFA-treated spheroids. (**E**) Captopril reverts the PLS poor-prognosis status in human liver fibrotic tissue precision-cut slices that were surgically resected from fibrotic patients diagnosed with alcoholic liver disease. Erlotinib was used as positive control. PLS reversal was determined by GSEA using vehicle-treated tissues as reference. Simplified heatmaps show (top) the classification of PLS status as poor (orange) or good (green) prognosis, and (bottom) the significance of induction (red) or suppression (blue) of poor- or good-prognosis genes. (**F**) Captopril decreases cell viability in a 3D patient-derived tumorspheroid model. HCC spheroids were generated from patient tumor tissues from different etiologies. Cell viability was assessed 3 days after treatment by measuring ATP levels. Each experiment shows mean ± SD in percentage compared with DMSO. For patient information, refer to Supplemental Table 4. Schemes of **D**–**F** were created with BioRender.com.

Finally, we validated the clinical relevance of the target pathways in liver fibrosis progression and hepatocarcinogenesis by expression studies in different clinical cohorts. GSEI analysis in a NAFLD/NASH patient cohort shows a significant enrichment of regulation of Ang levels in blood and of cell response to Ang, indicating that Ang signaling is associated with liver disease progression in metabolic liver disease (Figure 7A). Of note, an enrichment in the global RAS signature was also observed between healthy and patients with NASH (Figure 7A). Moreover, expression analyses in HCC patients revealed that ACE expression is increased in HCC induced by chronic HCV and HBV infection (Figure 7, B and C). Collectively these data indicate a potential functional role of the RAS also in viral hepatocarcinogenesis.

To obtain insights on whether captopril may have therapeutic efficacy in patients, we assessed the effects of captopril on the expression of the clinical PLS associated with HCC risk and survival in different patient-derived models. First, we applied a 3D multicellular spheroid model from patient tissues (including hepatocytes and nonparenchymal cells, NPCs) for NASH in which the 186 patient-derived PLS can be robustly induced by FFA exposure (Figure 7D) (13). We observed that captopril robustly reversed the poor-prognosis PLS induced by FFA (Figure 7D and Supplemental Table 3), suggesting that captopril treatment may be associated with therapeutic effect, reduced mortality, and HCC risk in patients. Corroborating these results, we applied a second model of precision-cut liver slices from fibrotic liver tissues preserving multi–cell type tissue architecture (10, 30). Captopril reversed the PLS poor-prognosis status with significantly decreased expression of the poor-prognosis PLS genes associated with HCC risk similarly to erlotinib, which is the EGFR inhibitor (Figure 7E). Together, these data indicate that captopril may have clinical efficacy in patients with advanced chronic liver disease by improving survival and decreasing HCC risk.

Aiming to study whether captopril exerts a direct anticancer effect also on established HCC, we applied another recently developed patient-derived 3D tumorspheroid model generated from tumor liver tissues, including cancer cells and the tumor microenvironment (12, 13). Sorafenib was used a control. As shown in Figure 7F, captopril slightly decreased cancer cell viability in patient-derived tumorspheroids, indicating that captopril may also have a direct effect on cancer arising in fibrotic and nonfibrotic liver disease (Figure 7F and Supplemental Table 4). The effect of captopril on tumorspheroids is independent from cancer etiology and patient treatment (Supplemental Table 4). Of note, mimicking suppression of the Ang signaling in cancer cells by inducing knockdown of the Ang receptor AGTR1 resulted in a decrease in cancer cell proliferation, explaining the effect of captopril on tumorspheroid system (Supplemental Figure 4). Collectively, these studies confirm the impact and translatability of the approach for patients with advanced liver disease and those at risk for HCC.

## Discussion

HCC chemoprevention is of vital importance, given the limited treatment options for liver cancer and the readily identifiable at-risk cirrhosis population. In this study, we identified captopril, an ACE inhibitor, as a generic compound preventing fibrotic liver disease progression toward HCC development. This conclusion is supported by the following findings: (a) ACE inhibitor captopril robustly and significantly inhibited fibrosis progression to HCC in 2 state-of-the-art animal models; (b) ACE and AGTR1 are overexpressed in animal models for liver disease and hepatocarcinogenesis, as well as patients with advanced liver disease progressing to HCC; (c) captopril reverts the induction of the poor-prognosis status of the PLS and of human cirrhosis modules robustly predicting HCC risk and survival in patients with advanced liver disease progressing to HCC; (d) captopril exhibited a direct anticancer effect in patient-derived HCC spheroids; and (e) crosstalk of the RAS with EGFR provides a mechanistic rationale for biological efficacy.

The systemic RAS is known to be a key regulator of blood pressure, sodium and water homeostasis, and response to tissue injury (14). In recent years, numerous studies have shown that the system is far more complex. Many organs, including heart, kidney, pancreas, and liver, locally express the RAS components, which regulate cell process such as cell growth, apoptosis, inflammation, and fibrogenesis (15, 16). The RAS pathway has been described to play a functional role in liver fibrosis (14, 15, 31–37). Different RAS inhibitors have been tested in a variety of animal models and have demonstrated antifibrotic effects (33, 36–42). However, their potential effect on HCC chemoprevention and its role in liver disease progression to cancer was unknown. Here, we show that the liver ACE is a safe and efficient target for HCC chemoprevention based on a large series of data across different systems, including patient-derived liver disease models. Interestingly, captopril was the ACE inhibitor with the highest efficacy to revert the poor-prognosis status of the PLS in our cell-based system (13).

Our mechanistic data show that crosstalk between the local RAS in the liver and the EGFR pathway in hepatocytes is most likely responsible for the HCC chemopreventive properties of captopril. Our study reveals that inhibition of the EGFR pathway by captopril treatment in vivo constitutes a potentially novel mechanism of action by which ACE inhibitors prevent HCC. Interestingly, our previous studies have shown that inhibition of EGFR by erlotinib effectively inhibits stellate cell activation, hepatic fibrosis, and development of HCC in animal models (18). In contrast to erlotinib, which is currently in clinical investigation for HCC chemoprevention (phase 2 clinical trial, NCT04172779), captopril has a superior safety profile.

Given the rising numbers of patients with advanced liver fibrosis and HCC risk driven by obesity, type 2 diabetes, and aging, there is a huge unmet medical need for HCC chemoprevention. Moreover, therapeutic approaches for HCC treatment are unsatisfactory and are frequently associated with severe side effects in patients (21). Given the proven safety profile in long-term administration (LiverTox: https://www.ncbi.nlm.nih.gov/books/NBK548504/), ACE inhibition may address a major unmet medical need by a simple and safe approach ready for clinical investigation. This concept is supported by retrospectives studies reporting a possible improvement of fibrosis by ACE inhibitors in patients with hepatitis C and patients with NASH without major safety issues (43–45). Other studies have shown that long-term exposure of patients with compensated liver cirrhosis (Child-Pugh class A) to ACE inhibitors does not increase the risk of end-stage renal disease (46). However, it should be noted that patients with decompensated liver cirrhosis (Child-Pugh classes B and C) are not ideal candidates for ACE inhibition due to significantly lower arterial blood pressure and increased risk of hepatorenal syndrome–associated renal dysfunction (14, 47, 48). Nevertheless, this limitation could be addressed by the development of liver-targeting ACE inhibitors for patients with chronic liver disease not tolerating ACE-inhibition. Taking this evidence into account, we suggest that captopril may be a chemopreventive drug of choice in patients with nondecompensated liver disease at risk for HCC.

Our data obtained in patient tissues, patient-derived models, and perturbation studies on the clinical PLS may indicate a therapeutic effect of an ACE-targeting agent on HCC chemoprevention. These findings are in line with retrospective studies showing that RAS inhibitors, alone or in combination with antiangiogenic drugs, reduce HCC risk and HCC recurrence and are associated with longer survival in HCC patients (49–51). Another recent study suggested that RAS inhibitors might prevent NAFLD development and progression in patients, supporting a protective role against cancer development (45). However, further investigation is need for arresting conclusions. Collectively, our data suggest that captopril is a simple, safe, and low-cost candidate approach for HCC chemoprevention ready for clinical investigation.

## Methods

Supplemental Methods are available online for further details about reagents, proteomics analyses, single-cell profiling, proteomic analysis, and Ang treatment.

### Human subjects.

Human liver tissues were obtained from liver disease patients undergoing liver resection with informed consent from all patients for deidentified use at the Center for Digestive and Liver Disease of the Strasbourg University Hospitals University of Strasbourg, France, or at Mount Sinai Hospital, New York, New York. All material was collected during a medical procedure strictly performed within the frame of the medical treatment of the patient. Informed consent is provided according to the Declaration of Helsinki. Detailed patient information and informed consent procedures are implemented by the Strasbourg University Hospital Biological Resources Center (HUS CRB). While there were clinical descriptive data available, the identity of the patients was protected by internal coding. A brief summary of patient characteristics (diagnosis and treatments) is provided in Supplemental Table 4.

The following public databases were used in the study are available on https://www.ncbi.nlm.nih.gov/geo/query (Figure 7, A and B): GSE48452, NASH patient cohort; GSE20140, HCV-HCC patient cohort; and GSE94660, HBV-HCC patient cohort.

### Research experiments on live vertebrates.

Eight-week-old male Wistar rats (Charles River Laboratories) received weekly i.p. injections of 50 mg/kg DEN to induce cirrhosis and liver cancer, or PBS, once per week over the course of 18 weeks. After 8 weeks, DEN-injured rats were randomly assigned to receive vehicle control (0.5% methylcelluose) (*n* = 10) or 20 mg/kg captopril (*n* = 10) by oral gavage daily for 9 weeks by a blinded technician. Livers were harvested and analyzed in week 18 (18). The sample size estimate was based on a *P* value of 0.01 at 95% power, assuming a 50% difference in means in tumor burden between control and drug-treated animals. In the NASH model of HCC, 8-week-old male Wistar rats (Charles River Laboratories) were fed either standard chow or CDAHFD consisting of (60 kcal% fat and 0.1% methionine by weight) for 18 weeks. After 6 weeks on CDAHFD, rats were randomly assigned to daily oral gavage of vehicle control (*n* = 8) or 20 mg/kg captopril (*n* = 8) to coincide with the onset of fibrosis. At the time of sacrifice, animals were anesthetized and sedated. A terminal blood collection was performed by cardiac puncture, and livers were removed for measurement of weight, snap frozen for analysis, or fixed in formalin for histology.

### Cells.

Huh7.5.1 were a gift from F. Chisari (The Scripps Research Institute, La Jolla, San Diego, California, USA). LX2 were purchased from Merck. THP1 were purchased from ATCC. Huh7.5.1 and LX2 cells were cultured in DMEM (Thermo Fisher Scientific) supplemented with 10% heat-decomplemented FBS, gentamycin (0.05 mg/mL), and nonessential amino acids (complete DMEM) at 37°C with 5% CO_2_. Cell lines were certified mycoplasma free. For proliferation arrest and differentiation (Huh7.5.1^dif^ cells), Huh7.5.1 cells were cultured in complete DMEM containing 1% DMSO. THP1 cells were cultured and differentiation in RPMI 1640 medium with GlutaMAX-I supplement and HEPES, and they were supplemented with 10% FBS and gentamycin (0.05 mg/mL) (Thermo Fisher Scientific). To generate THP-1–derived macrophages (M0), cells were treated with PMA (320 nM) (Promega) for 48 hours. For coculture experiment, Huh7.5.1 were cultured with 20% LX2 or 20% LX2 and 10% macrophages in complete DMEM for 3 days before treatment.

### HCV infection of Huh7.5.1^dif^ cells.

Cell culture–derived HCVcc Jc1 (genotype 2a/2a) were produced in Huh7.5.1 cells as described (52). HCVcc infectivity was determined by calculating the TCID_50_ as described (53). Huh7.5.1^dif^ cells were infected with HCV Jc1 for the indicated time points. Cell culture supernatants from mock-electroporated cells were used for control experiments. HCV infection was assessed by quantitative PCR (qPCR) of intracellular HCV RNA. For PLS assays, cells were treated with captopril (5 μM), erlotinib (1 μM), tipifarnib (10 μM), or Fr180204 (10 μM) 7 days after infection for 3 more days.

### FAA treatment.

Huh7.5.1^dif^ cells were cocultured with 20% LX-2 stellate cells for 3 days in DMEM supplemented with 10% heat-decomplemented FBS, gentamycin, and 1% DMSO at 37°C and 5% CO_2_. Cells were then incubated with FFA (800 μM oleic acid and 400 μM palmitic acid) for 72 hours.

### PLS calculation.

The PLS 186 (complete) or 32 gene (reduced, see below) expression profiling was performed using 250–500 ng total RNA by using nCounter Digital Analyzer system (NanoString). For full PLS gene list, refer to Supplemental Table 1. PLS gene expression was normalized according to the gene expression of 6 housekeeping genes using GenePattern genomic analysis toolkits (54, 55). Induction or suppression of the PLS signature was determined as previously reported by using GSEA, implemented in GenePattern genomic analysis toolkits (54, 55). PLS was always determined by using control (CTRL) cells, CTRL animals, or CTRL patient–derived tissues as references. Results are presented as simplified heatmaps showing the classification of PLS global status as poor or good prognosis and the significance of induction/suppression of PLS genes (log_10_ of FDR values). Global status corresponds to the difference between low-risk and high-risk gene expression. For discovery in cell culture, the results are considered as significant if FDR < 0.25 according to GSEA. For validation in vivo and in ex vivo models, the results are considered as significant if FDR < 0.05 (56). The 32-gene signature is a reduced version of the PLS, comprising gene bioinformatically defined and validated in multiple patient cohorts in previous studies (9, 10). The gene signature was bioinformatically reduced from 186 genes to 32 genes and implemented in an FDA-approved diagnostic assay platform specifically designed for clinical use (57–60).

### RNA-Seq on rat liver tissues.

Total RNA was isolated from snap-frozen liver tissues of PBS-treated control rats, DEN-treated cirrhotic rats, and DEN-treated rats with captopril treatment (*n* = 3 for each experimental group) using RNeasy kit (Qiagen). After quality assessment (RNA integrity score > 9), 200 ng total RNA samples were used for library preparation using Tru-Seq kit (Illumina) and sequenced on NextSeq 550 genome sequencer (Illumina) according to manufacturer’s instruction to generate 100 nt single-end RNA-Seq reads. Raw reads were aligned to the reference genome (rattus norvegicus, Rnor_6.0) using the spliced gap aligner STAR (61), and count-based quantitation was carried out by the Subread package featureCounts at the gene level based on ENSEMBL annotation build (Rnor_6.0.101). The whole-genome transcriptome read count data were normalized and modeled with overdispersed Poisson data as trimmed mean of M values (TMM) using a negative binomial model in the Bioconductor package edgeR (62). For subsequent data analysis, genes with no expression in more than 50% of the samples and low variance across the samples (coefficient of variance < 0.01) were excluded. The rat genes were mapped onto human orthologues based on NCBI Homologene database (build 68, https://www.ncbi.nlm.nih.gov/homologene), and expression levels of multiple rat genes mapped to a human gene were summarized with their median value. Dysregulation and modulation of molecular pathways were assessed by GSEA (56) using the Hallmark gene sets (63) from Molecular Signature Database (MSigDB) v.7.0 (64). Molecular pathways dysregulated in comparison between the DEN rats and the control rats were selected at a significance cutoff of FDR < 0.005 and visualized as GSEI, defined as –log_10_ (GSEA *P* value based on 1000 gene permutations), with a sign of GSEA normalized enrichment score (NES) as well as a bar plot of NES. We similarly analyzed human cirrhosis coregulatory gene modules defined in our previous study (10) and EGFR transcriptional target gene signatures from the MSigDB database. The transcriptome data set is available via NCBI Gene Expression Omnibus (https://www.ncbi.nlm.nih.gov/geo/; accession no. GSE157919). All bioinformatics data analyses were performed by using GenePattern genomic analysis toolkit (www.genepattern.org) or R statistical package (www.r-project.org).

### Culture of organotypic ex vivo patient liver slice, patient-derived spheroids, and tumorspheroids. Organotypic liver slices.

Fresh liver tissue sections (300 μm thick) were made from surgically resected fibrotic livers from liver disease patients using Krumdieck Tissue Slicer MD6000 (Alabama Research and Development) (10). The tissues were cultured with captopril (100 μM), erlotinib (5 μM), or DMSO vehicle control for 48 hours and harvested for gene expression analysis as described above. For ex vivo tissue culture, we used a higher concentration compared with culture of cell lines according to ref. 65.

### Patient-derived spheroids.

Spheroids were generated from liver tissues from patients without liver disease undergoing liver resection for metastasis of colorectal cancer. Tissues were perfused and dissociated as described (13). Total cell population including parenchymal and NPCs was used to generated multicellular spheroids in Corning 96-well Black/Clear Bottom Low Flange Ultra-Low Attachment Microplate (Corning) (13). Spheroids were then treated with FFA and/or captopril (20 μM) for a total of 3 days before PLS assessment. DMSO was used as negative control.

### Patient-derived tumorspheroids.

Tumorspheroids were generated from patient HCC liver tissues undergoing surgical resection and dissociated using Human Tumor Dissociation Kit as described (Miltenyi Biotec) (13). Total cell populations, including parenchymal cells and NPCs, were used to generated multicellular tumorspheroids as described above. After 48 hours, HCC-derived spheroids were treated with captopril at 20 μM and sorafenib at 1 μM as a reference CTRL or DMSO vehicle control for 4 days. Fresh medium containing DMSO or drugs were added every day. Cell viability was assessed using CellTiter-Glo Luminescent Cell Viability Assay (Promega), according to manufacturer’s instruction. For spheroids and tumorspheroids, patient information is summarized in Supplemental Table 4.

### Statistics.

In vitro experiments were reproduced 2 (PLS) or 3 times in an independent manner with similar results. The precise number (*n*) of biologically independent samples used to derive statistics is indicated in the figure legends. The data are presented as the mean ± SD (unless otherwise stated) and were analyzed by the unpaired 2-tailed Student’s *t* test or the 2-tailed Mann-Whitney *U* test, as indicated in figure legends, after determination of distribution by the Shapiro-Wilk normality test. *P* < 0.05 was considered statistically significant. Significant *P* values are indicated by asterisks in the individual figures. Statistical analyses were performed with GraphPad Prism 8 software. No statistical analyses were performed if *n* < 4. For in vivo experiments, the sample size estimate was based on a *P* value of 0.01 at 90% power assuming a 50% difference in means in tumor burden with 33% SD between control and drug-treated animals. The Kruskal-Wallis test, followed by Dunn’s multiple comparisons test, was used to compare the 3 groups or unpaired Student’s *t* test to compare 2 groups. *P* < 0.05 was considered statistically significant. For the PLS assay, variation of the poor- and the good-prognosis genes was determined by a NES obtained using GSEA. Significance of the data was determined by the FDR values. According to GSEA (https://www.gsea-msigdb.org/gsea/index.jsp), results are significant if FDR < 0.25 for discovery in cell culture. Results are expressed as a heatmap (log _10_ of FDR). For RNA-Seq on liver tissues, the threshold was adjusted at FDR < 0.05. For clinical data presented in Figure 7, data were extracted from a publicly available database (https://www.ncbi.nlm.nih.gov/geo/). GEO number is indicated in each figure panel. For patient-derived spheroids, due to the rarity and the limited quantity of patient liver tissues, the experiments were performed only 1 time in quadruplicate but on several patients. For cell culture/in vitro data, poor or insufficient technical quality of experiment or data analysis resulted in exclusion of samples (also known as the empirical method, in which values are excluded if X < or > to μ ± σ).

### Study approval.

The protocols for experiments with human tissues were approved by the local Ethics Committee of the University of Strasbourg Hospitals and Mount Sinai Hospital, respectively (Center for Digestive and Liver Disease of the Strasbourg University Hospitals University of Strasbourg, France: DC-2016-2616 and RIPH2 LivMod IDRCB 2019-A00738-49, ClinicalTrials.gov NCT04690972; Mount Sinai Hospital, New York City, NY: HS13-00159). All animals were housed in accordance with the guidelines of the Massachusetts General Hospital IACUC (protocol approval nos. 2007N000113 and 2009N000207) and received humane care according to the criteria outlined in the *Guide for the Care and Use of Laboratory Animals* (National Academies Press, 2011).

## Author contributions

TFB initiated and coordinated the study. TFB, YH, and BCF conceived the project. EC, SL, MS, SB, NF, SCB, ES, MAO, CP, SCD, SG, GA, NS, NVR, and JL designed and performed experiments and analyzed data. NF, HES, SZ, TQ, and FAR performed PCLS experiments and analysis and bioinformatic analyses of RNA-Seq data. RTC critically advised and commented for the project. NP performed the computational analyses of scRNA-Seq profiling. EF and PP provided patient-derived tissues. FG, FDZ, and CS provided clinical data and information. EC, YH, BCF, and TFB wrote the manuscript and prepared the figures. KKT and BCF supervised animal experiments. YH supervised the bioinformatic analyses and liver precision-cut slice experiments.

## Supplementary Material

Supplemental data

## Version 1 07/08/2022

Electronic publication
