# Supplementary material for: Hepatocellular carcinoma chemoprevention by targeting the angiotensin-converting enzyme and EGFR transactivation
Source: JCI Insight. 2022 Jul 8;7(13):e159254. doi: 10.1172/jci.insight.159254 (PMC9310532; doi:10.1172/jci.insight.159254)
Supplement: Supplemental data [file jciinsight-7-159254-s187.pdf]

Hepatocellular carcinoma chemoprevention by targeting the angiotensin converting enzyme and EGFR transactivation

SUPPLEMENTAL MATERIAL AND METHODS

Reagents and antibodies. DMSO, oleic acid, palmitic acid, angiotensin I, angiotensin II, and recombinant EGF were purchased from Sigma-Aldrich; Fr180204 from Merck; captopril and tipifarnib from Selleckchem. The Human Phospho-RTK Array kit and the Proteome Profiler Human Phospho-kinase Array kit were obtained from R&D Systems. PMA was purchased from Promega and IL4 and IL13 from Bio-Techne. The antibodies utilized are described in the following table:

Target	Catalogue number	Supplier	Method	Antibody concentration
ACE	ab124734	Abcam	WB	1 to 1000
ACTIN	ab8224	Abcam	WB	1 to 10000
PCNA	2586	Cell Signaling	IHC	1 to 500
pERK	AF1018	R&D	WB	1 to 1000
ERK	MAB1576	R&D	WB	1 to 1000
p-JNK	9251	Cell Signaling	WB	1 to 1000
JNK	9252	Cell Signaling	WB	1 to 1000
p-P38	9211	Cell Signaling	WB	1 to 1000
P38	9212	Cell Signaling	WB	1 to 1000

Perturbation studies using angiotensin I and II (Ang I, Ang II) treatment. To study the effect of Ang I and Ang II on the PLS induction, Huh7.5.1^{dif} cells were grown in complete DMEM supplemented with only 1% FBS and treated with Ang I or Ang II (10 µM each) for 3 days before to assess the PLS. As the half-life of these molecules is short, cells were treated every 12h. To study the effect of Ang I and Ang II on EGFR activation, Huh7.5.1^{dif} cells were grown in 1% FBS medium for 24h and in serum-free medium 1h before the incubation with angiotensins, or EGF (100 ng/mL) as a positive CTRL for 30 min at 37°C prior to cell lysis.

Single cell RNA-Seq profiling. Huh7.5.1^{dif} cells were infected with highly purified Flag tagged virus, HCV Jc1E2^{FLAG} ($TCID_{50} = 7 \times 10^5$/mL) as described (1). On day 7, single cells were sorted into 96-well plates. Cellular mRNA was isolated and analyzed as described (2, 3). HCV RNA was co-amplified with cellular mRNA using a SMART-compatible primer (5'-Biotin-AAGCAGTGGTATCAACGCAGAGTACTCTGCGGAACCGGTGAGTA-3') derived from (4). For all single cells, reads were aligned to the human hg19 UCSC reference as well as the HCV Jc1 reference using Tophat (5, 6) and gene expression levels were quantified for 21948 human genes using Cuffquant from Cufflinks (5–7). For each of the single cells we estimated the HCV viral load, which we defined as the percentage of mapped viral read pairs relative to the total number of mapped human and viral read pairs, thus the percentage of HCV viral reads. Enrichment of EGFR and MAPK signatures in association with the HCV viral load in single cell RNA-Seq data was tested using the pre-ranked GSEA module in GenePattern with Pearson correlation as the rank metric. Differential expression z-scores of captopril treatment (using signature CPD001_MCF7_6H:BRD-K54529596-001-19-8:10) were downloaded from the LINCS database. Probes were collapsed to unique genes by prioritizing landmark over inferred probes and selecting the probe with the highest absolute z-score in case of multiple probes. These z-scores were used as rank metric in a pre-ranked GSEA analysis to test enrichment of EGFR and MAPK pathways in response to captopril treatment. Significance of the overlap between the EGFR/MAPK pathway leading-edge genes (the core of a gene set that accounts for the enrichment signal (8)) that are modulated by both captopril treatment and HCV infection was determined using the hypergeometric test statistic (with N equals to the number of genes in each signature). Expression heatmaps were visualized using GENE-E (www.broadinstitute.org/GENE-E). All genomic datasets used for this study are available at NCBI Gene Expression Omnibus database (www.ncbi.nlm.nih.gov/geo, accession number: GSE66843).

Relative Quantification (RQ) for qRT-PCR experiments on rat liver tissues. RQ is calculated using the difference in Cq as a determinant of the differences in concentration of the target sequence in experimental samples relative to the control samples (PBS or Normal chow). The 18S ribosomal RNA was used as an internal control to normalize the target Cq.

Proteomic analyses. Phosphorylation of RTK and intracellular kinases were studied using the Human Phospho-RTK Array Kit and Proteome Profiler Human Phospho-kinase Array, respectively, according to manufacturer's instructions (R&D Systems). The relative dot-blot density of the phosphorylated proteins in samples compared to controls was quantified using Image J software (NIH) by elliptical selection of individual dots and measuring standard deviation and integrated density. To study the impact of HCV infection on RTK activation, Huh7.5.1^{dif} cells were infected with HCV Jc1E2^{FLAG} for 7 days and lysed using Lysis Buffer 17 (R&D systems). To assess the effects of captopril on cell signaling, Huh7.5.1^{dif} cells were infected with HCV Jc1 for 7 days and treated with captopril (1 µM), for 3 additional days prior to cell lysis.

Macrophages M1 and M2 phenotype.

To generate THP-1-derived macrophages (M0), cells were treated with PMA (320 nM) for 48 hours. To induce M1 phenotype, M0 macrophages were treated for 24h with LPS (100 ng/mL) and IFNγ (20 ng/mL). To induce M2 phenotype, M0 macrophages were treated for 24h with IL4 and IL4 (20 ng/mL).

SUPPLEMENTAL FIGURES

Supplemental figure 1:

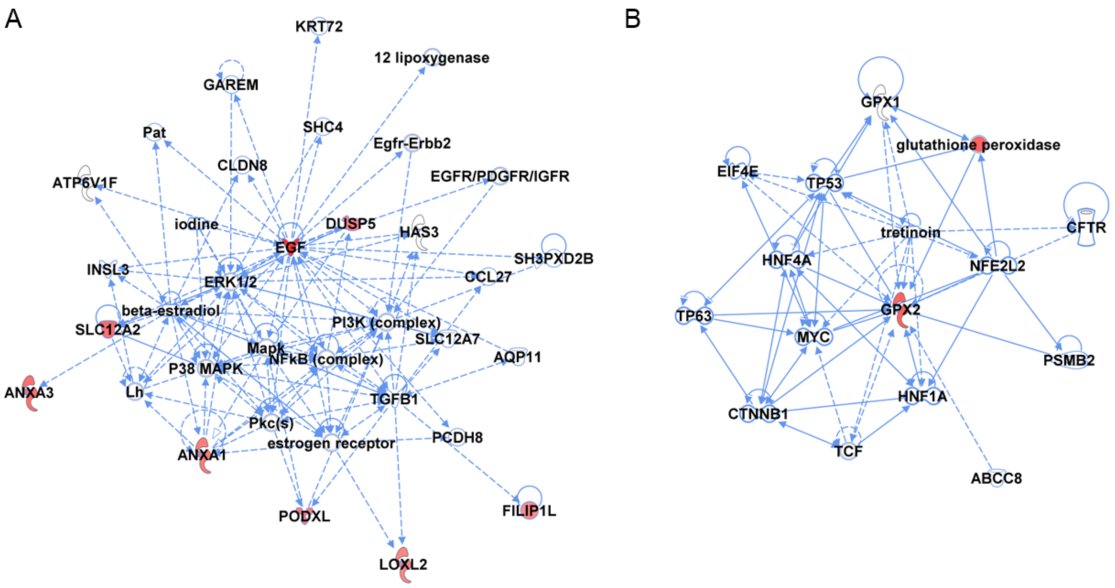

Supplemental figure 1: Co-regulated gene networks in PLS high-risk genes common to HCV- and HBV-related disease and alcoholic liver disease (Ingenuity Pathway Analysis). Expression of the PLS was analyzed in clinical liver tissues from HCV- and HBV-related liver disease and alcoholic liver disease. The PLS high-risk genes tFighat were commonly modulated in clinical samples as core enriched genes in GSEA were then analyzed by Ingenuity Pathway Analysis tools as described in Methods. A fibrosis-related network including the EGFR/MAPK signaling pathway (**A**) and an oncogenic network including the p53/Myc pathway (**B**) are shown. The PLS high-risk genes included in networks are in red. Solid and dashed lines indicate direct and indirect interactions, respectively

Supplemental figure 2:

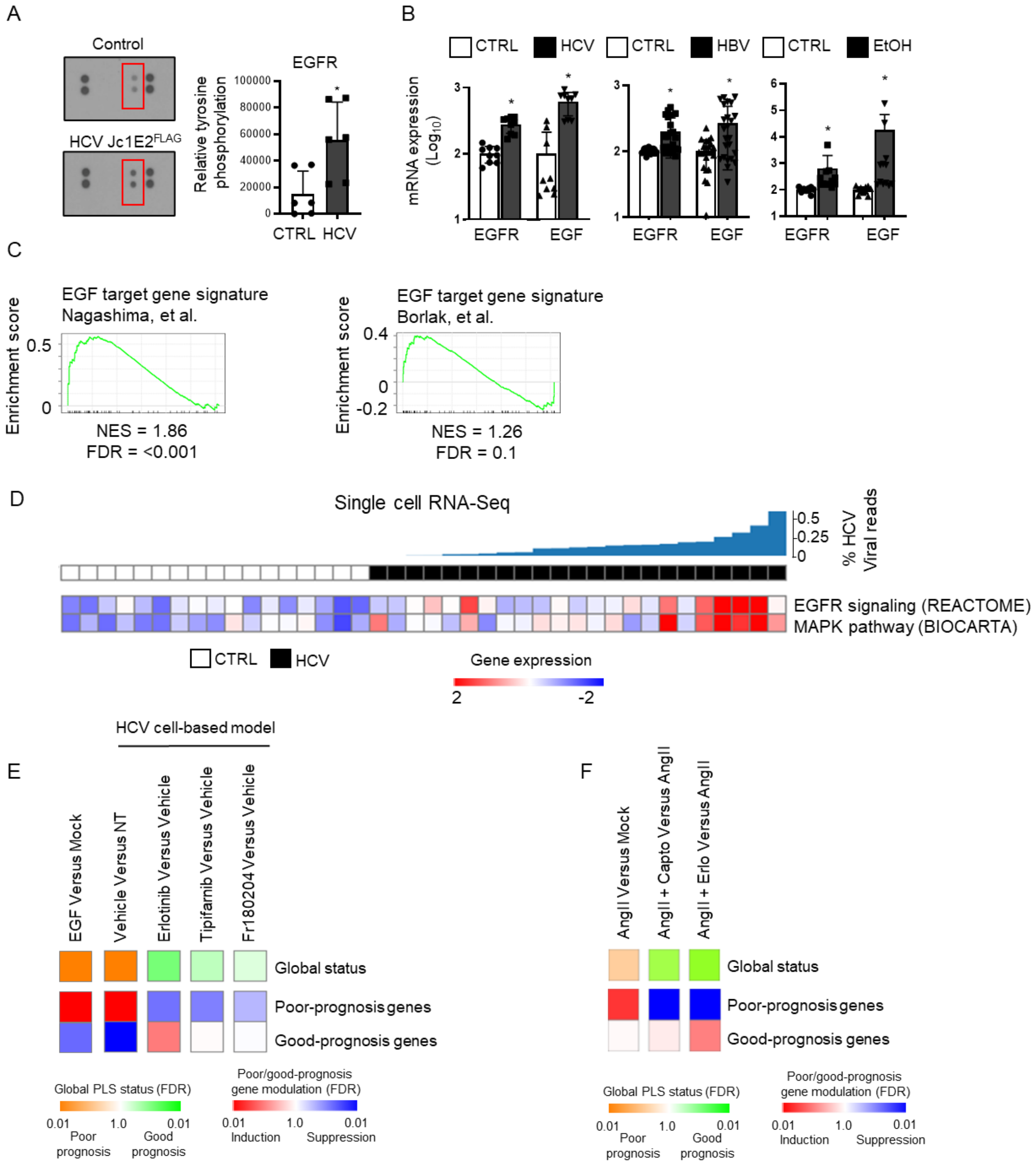

Supplemental 2: EGFR is a pan-etiology driver of the PLS in the cell-based system A-B. Persistent HCV infection of Huh7.5.1^{dif} cells increases EGFR phosphorylation. (A) Phospho-array analyses. One representative experiment out of three is shown. Quantification of dot blot intensities show the mean ± s.e.m. of integrated dot blot densities from three independent experiments performed in duplicate. * = $p < 0.05$ (Mann-Whitney U test). (B) HCV-mediated signaling induces EGF target gene signatures in Huh7.5.1^{dif} cells (GSEA analysis). **C**. EGFR and EGF mRNA expression in HCV-infected Huh7.5.1^{dif} cells (n = 9); HBV-infected HepG2-NTCP cells (n = 9); and Huh7.5.1^{dif} cells incubated in presence of 40 mM ethanol (n = 12). Mean percentage of control ± s.e.m. is shown. ** = $p < 0.01$ (Mann-Whitney U test). **D.** Single cell RNA-Seq profiling reveals virus-dependent induction of the EGFR and MAPK pathways in HCV-infected Huh7.5.1^{dif} cells. Heatmaps show modulation of EGFR/MAPK pathway (mean expression of leading-edge genes in single-sample GSEA standardized row-normalized as Z-score). Results are ordered by infection status (white: non-infected cells, n = 17; black: infected cells, n = 23), and by increasing HCV viral load (blue bar). **E.** Small molecule inhibitors reverse expression of the poor-prognosis PLS. PLS induction was determined by GSEA analysis using “Mock” non infected cells as reference. Simplified heatmaps show: (top) the classification of PLS status as poor (orange) or good (green) prognosis; (bottom) the significance of induction (red) or suppression (blue) of poor- or good-prognosis genes. **F.** Erlotinib and captopril reversed the Ang II-induced poor-prognosis PLS. PLS induction was determined by GSEA analysis using “Mock” non treated cells as reference. FDR: false discovery rate. NES: normalized enrichment score.

Supplemental figure 3:

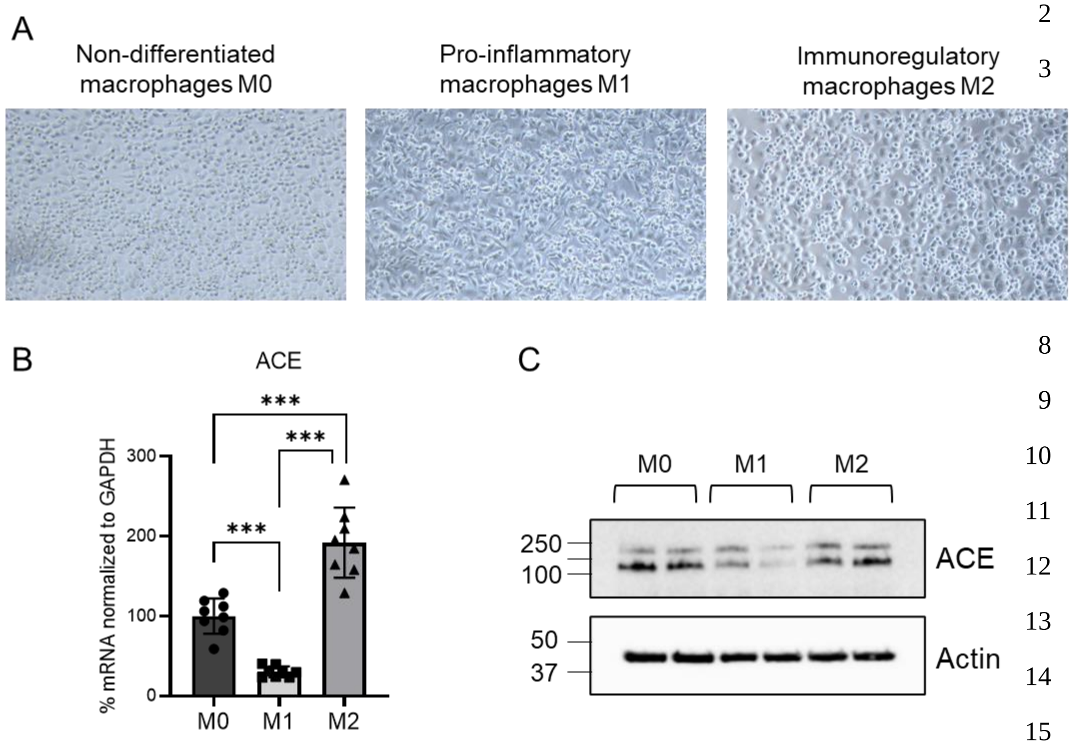

Supplemental figure 3: ACE is highly expressed in M2 macrophages. A. THP1 were PMA-differentiated in M0 macrophages. M1 pro-inflammatory or M2 immunoregulatory phenotypes were induced by treatment with LPS and IFNγ or with IL13 and IL4 respectively. Representative pictures are shown. **B.** ACE is highly expressed in M2 immunoregulatory macrophages. ACE expression was determined by qRT-PCR. Results are from three independent experiments (n= 8) (% mean ± s.d) *** $p < 0.001$, One-way ANOVA followed by Tukey's multiple comparisons test. **C.** ACE protein expression in M0, M1 and M2 macrophage is shown. One representative experiment out 2 is shown.

Supplemental figure 4:

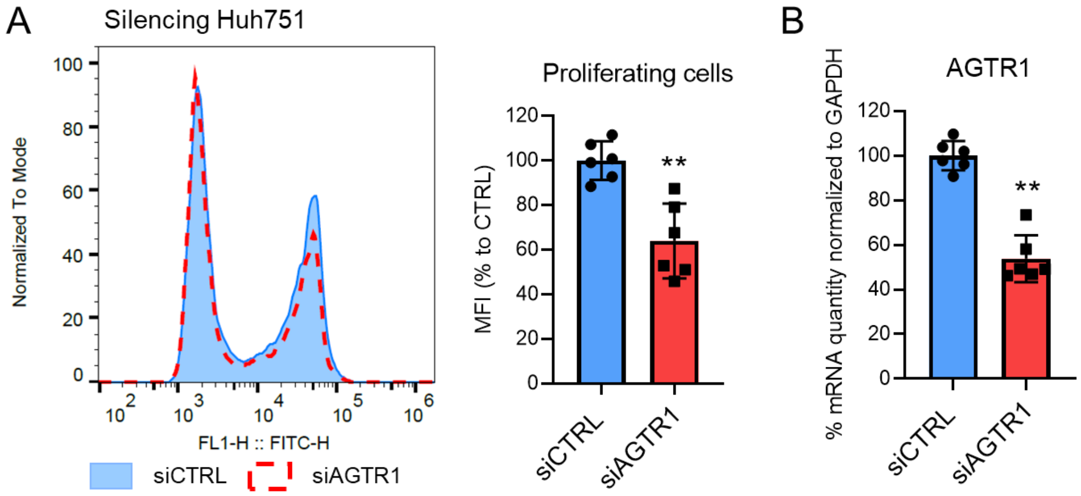

Supplemental figure 4: AGTR1 knock down decreases cancer cell proliferation. A. Effect of AGTR1 on Huh7.5.1 cancer cell proliferation assessed by EdU-incorporation assay (flow cytometry). Graph show % +/- s.d. of MFI (siCTRL = 100%) from 3 independent experiments performed in duplicate in cell transfected with siCTRL and siAGTR1. **B.** siRNA efficacy was assessed by qRT-PCR. ** $p < 0.01$, two-tailed Mann-Whitney U test.

SUPPLEMENTAL TABLES

SUPPLEMENTAL TABLE 1

Poor-prognosis genes

Gene ID	Gene Symbol	Description
**3983**	**ABLIM1**	**actin binding LIM protein 1**
10097	ACTR2	ARP2 actin-related protein 2 homolog (yeast)
120	ADD3	adducin 3 (gamma)
165	AEBP1	AE binding protein 1
11214	AKAP13	A kinase (PRKA) anchor protein 13
301	ANXA1	annexin A1
306	ANXA3	annexin A3
162	AP1B1	adaptor-related protein complex 1, beta 1 subunit
496	ATP4B	ATPase, H+/K+ exchanging, beta polypeptide
596	BCL2	B-cell CLL/lymphoma 2
8030	CCDC6	coiled-coil domain containing 6
6363	CCL19	chemokine (C-C motif) ligand 19
6366	CCL21	chemokine (C-C motif) lignad 21
962	CD48	CD48 molecule
10523	CHERP	calcium homeostasis endoplasmic reticulum protein
22856	CHSY1	carbohydrate (chondroitin) synthase 1
1307	COL16A1	collagen, type XVI, alpha 1
1282	COL4A1	collagen, type IV, alpha 1
**1293**	**COL6A3**	**collagen, type VI, alphae 3**
1359	CPA3	carboxypeptidase A3 (mast cell)
1501	CTNND2	catenin (cadherin-associated protein), delta 2 (neural plakophilin-related arm-repeat protein)
7852	CXCR4	chemokine (C-X-C motif) receptor 4
1601	DAB2	disabled homolog 2, mitogen-responsive phosphoprotein (Drosophila)
780	DDR1	discoidin domain receptor family, member 1

1847	DUSP5	dual specificity phosphatase 5
**9170**	**EDG4**	**endothelial differentiation lysophosphatidic acid G-protein-coupled**
**1950**	**EGF**	**epidermal growth factor (beta-urogast)**
54898	ELOVL2	elongation of very long chain fatty acids (FEN1/Elo2, SUR4/Elo3, yeast)-transcription factor 4
2013	EMP2	epithelial membrane protein 2
**2043**	**EPHA4**	**EPH receptor A4**
9852	EPM2AIP1	EPM2A (laforin) interacting protein 1
2200	FBN1	fibrillin 1
11259	FILIP1L	filamin A interacting protein 1-like
**2326**	**FMO1**	**flavin containing monooxygenase 1**
**2488**	**FSHB**	**follicle stimulating hormone beta polypeptide**
2629	GBA	glucosidase, beta; acid (includes glucosylceramidase)
**2877**	**GPX2**	**glutathione peroxidase 2 (gastrointestinal)**
9734	HDAC9	histone deacetylase 9
10362	HMG20B	high-mobility group 20B
22858	ICK	intestinal cell (MAK-like) kinase
8870	IER3	immediate early response 3
10437	IFI30	interferon gamma-inducible protein 30
3489	IGFBP6	insulin-like growth factor binding protein 6
8826	IQGAP1	IQ motif containing GTPase ctivating protein 1
**3680**	**ITGA9**	**integrin, alpha 9**
3855	KRT7	keratin 7
4017	LOXL2	lysyl oxidase-like 2
4026	LPP	LIM domain containing preferred translocation partner in lipoma
**4316**	**MMP7**	**matrix metallopeptidase 7 (matrilysin, uterine)**
**23397**	**NCAPH**	**non-SMC condensin I complex, subunit H**
4791	NFKB2	nuclear factor of kappa light polypeptide gene enhancer in B-cells 2
51406	NOL7	nucleolar protein 7, 27kDa
**4843**	**NOS2A**	**nitric oxide synthase 2A (inducible, hepatocytes)**
**4922**	**NTS**	**neurotensin**

5420	PODXL	podocalyxin-like
**5593**	**PRKG2**	**protein kinase, cGMP-dependent type II**
5698	PSMB9	proteasome (prosome, macropain) subunit, beta type 9 (large multifunctional peptidase 2)
**23029**	**RBM34**	**RNA binding motif protein 34**
6035	RNASE1	ribonuclease, Rnase A family 1 (pancreatic)
**5055**	**SERPINB2**	**serpin peptidase inhibitor, clade B (ovalbumin), member 2**
5271	SERPINB8	serpin peptidase inhibitor clade B (ovalbumin), member 8
**6456**	**SH3GL2**	**SH3-domain GFB2-like 2**
6558	SLC12A2	solute carrier family 12 (sodium/potassium/chloride transporters), member
6541	SLC7A1	solute carrier family 7 (cationic amino acid transporter, y+ system)
6586	SLIT3	slit homolog 3 (Drosophila)
**6672**	**SP100**	**SP100 nuclear antigen**
6925	TCF4	transcription factor 4
7004	TEAD4	TEA domain family member 4
7041	TGFB1I1	transforming growth factor beta 1 induced transcript 1
10188	TNK2	tyrosine kinase, non-receptor, 2
**7204**	**TRIO**	**triple functional domain (PTPRF interacting)**
9819	TSC22D2	TSC22 domain family, member 2
7456	WIPF1	WAS/WASL interacting protein family, member 1

Good-prognosis genes

16	AARS	alanyl-tRNA synthetase
10965	ACOT2	acyl-CoA thioesterase 2
**6296**	**ACSM3**	**acyl-CoA synthetase medium-chair family member 3**
128	ADH5	alcohol dehydrogenase 5 (class III), chi polypeptide
130	ADH6	alcohol dehydrogenase 6 (class V)
**151**	**ADRA2B**	**adrenergic, alpha-2B- receptor**
10327	AKR1A1	aldo-keto reductase family 1, member A1 (aldehyde reductase)
6718	AKR1D1	aldo-keto reductase family 1, member D1 (delta 4-3 ketosteroid-5-beta)
211	ALAS1	aminolevulinate, delta-synthase 1

**223**	**ALDH9A1**	**aldehyde dehydrogenase 0 family, member A1**
157567	ANKRD46	ankyrin repeat domain 46
316	AOX1	aldehyde oxidase 1
367	AR	androgen receptor (dihydrotestosterone receptor, testicular feminization; spinal and bulbar muscular atrophy; Kennedy disease)
378	ARF4	ADP-ribosylation factor 4
417	ART1	ADP-ribosyltransferase 1
27163	ASAHL	N-acylsphingosine amidohydrolase (acid ceramidase)-like
27032	ATP2C1	ATPase, Ca++ transporting, type 2C, member 1
513	ATP5D	ATP synthase, H+ transporting, mitochondria F1 complex, delta subunit
10159	ATP6AP2	ATPase, H+ transporting, lysosomal accessory protein 2
10458	BAIAP2	BAI1-associated protein 2
25874	BRP44	brain protein 44
725	C4BPB	complement component 4 binding protein, beta
727	C5	complement component 5
732	C8B	complement 8, beta polypeptide
735	C9	complement component 9
799	CALCR	calcitonin receptor
10694	CCT8	chaperonin containing TCP1, subunit 8 (theta)
1369	CPN1	carboxypeptidase N, polypeptide 1
1371	CPOX	coproporphyrinogen oxidase
1385	CREB1	cAMP responsive element binding protein 1
1486	CTBS	chitobiase, di-N-acetyl-
23316	CUTL2	cut-like 2 (Drosophila)
1528	CYB5A	cytochrome b5 type A (microsomal)
1555	CYP2B6	cytochrome P450, family 2, subfamily B, polypeptide 6
1603	DAD1	defender against cell death 1
22839	DLGAP4	discs, large (Drosophila) homolog-associated protein 4
9732	DOCK4	dedicator of cytokinesis 4
667	DST	dystonin
1967	EIF2B1	eukaryotic translation initiation factor 2B, subunit 1 alpha, 26kDa

2010	EMD	emerin (Emery-Dreifuss dystrophy)
2073	ERCC5	excision repair cross-complementing rodent repair deficiency, complementation group 5
2158	F9	coagulation factor IX (plasma thromboplastic component, Chistmas disease, hemophilia B)
116496	FAM129A	family with sequence similarity 129, member A
2642	GCGR	glucagon receptor
2646	GCKR	glucokinase (hexokinase 4) regulator
2677	GGCX	gamma-glutamyl carboxylase
2690	GHR	growth hormone receptor
2705	GJB1	gap junction protein, beta 1, 32kDa
2868	GRK4	G protein-coupled receptor kinase 4
2915	GRM5	glutamate receptor, metabotropic 5
2944	GSTM1	glutathione S-transferase M1
23498	HAAO	3-hydroxyanthranilate 3,4-dioxygenase
3026	HABP2	hyaluronan binding protein 2
3155	HMGCL	3-hydroxymethyl-3-methylglutaryl-Coenzyme A lyase (hydroxymethylglutaricaciduria)
3156	HMGCR	3-hydroxy-3-methylglutaryl-Coenzyme A reductase
11145	HRASLS3	HRAS-like suppressor 3
3336	HSPE1	heat shock 10kDa protein 1 (chaperonin 10)
3479	IGF1	insulin-like growth factor 1 (somatomedin C)
**3612**	**IMPA1**	**inositol(myo)-1(or 4)-monophosphatase 1**
3642	INSM1	insulinoma-associated 1
3760	KCNJ3	potassium inwardly-rectifying channel, subfamilu J, member 3
3990	LIPC	lipase, hepatic
51237	MGC29506	NA
2956	MSH6	mutS homolog 6 (E. coli)
79731	NARS2	asparaginyl-tRNA synthetase 2, mitochondria (putative)
29937	NENF	neuron derived neutrophic factor
5105	PCK1	phosphoenolpyruvate carboxykinase 1 (soluble)
5833	PCYT2	phosphate cytidylyltransferase 2, ethanolamine
**5207**	**PFKFB1**	**6-phosphofructo-2-kinase/fructose-2,6-biphosphatase 1**

10026	PIGK	phosphatidylinositol glycan anchor biosynthesis class K
**5313**	**PKLR**	**pyruvate kinase, liver and RBC**
5331	PLCB3	phospholipase C, beta 3 (phosphatidylinositol-specific)
5336	PLCG2	phospholipase C, gamma 2 (phosphatidylinositol-specific)
5340	PLG	plasminogen
5372	PMM1	phosphomannomutase 1
5442	POLRMT	polymerase (RNA) mitochondria (DNA directed)
5446	PON3	paraoxonase 3
**5502**	**PPP1R1A**	**protein phosphatase 1, regulatory (inhibitor) submunit 1A**
5627	PROS1	protein S (alpha)
**5691**	**PSMB3**	**proteasome (promosome, macropain) subunit, beta type, 3**
26469	PTPN18	protein tyrosine phosphatase, non-receptor type 19 (brain-derived)
**5771**	**PTPN2**	**protein tyrosine phosphatase, non-receptor type 2**
5893	RAD52	RAD52 homolog (S. cerevisiae)
5982	RFC2	replication factor C (activator 1) 2, 40kDa
**6018**	**RLF**	**rearranged L-myc fusion**
**9252**	**RPS6KA5**	**ribosomal protein S6 kinase, 90 kDa, polypeptide 5**
6240	RRM1	ribonucleotide reductase M1 polypeptide
6309	SC5DL	sterol-C5-desaturase (ERG3 delta-5-desaturase homolog, S. cerevisiae)
6447	SCG5	secretogranin V (7B2 protein)
6391	SDHC	succinate dehydrogensase complex, subunit C, integral membrane protein
8991	SELENBP1	selenium binding protein 1
6427	SFRS2	splicing factor, arginine/serin-rich 2
2542	SLC37A4	solute carrier family 37 (glucose-6-phosphate transporter), member 4
8671	SLC4A4	solute carrier family 4, sodium bicarbonate cotransporter member 4
29887	SNX10	sorting nexin 10
6721	SREBF2	sterol regulatory element binding transcription factor 2
6744	SSFA2	sperm specific antigen 2
8802	SUCLG1	succinate-CoA ligase, GDP-forming, alpha subunit
9013	TAF1C	TATA box binding protein (TBP)-associated factor, RNA polymerase I, C, 1110kDa

6999	TDO2	tryptophan 2,3-dioxygenase
1678	TIMM8A	translocase of inner mitochondria membrane 8 homolog A (yeast)
7108	TM7SF2	transmembrane 7 superfamily member 2
**27346**	**TMEM97**	**transmembrane protein 97**
7189	TRAF6	TNF receptor-associated factor 6
**7276**	**TTR**	**transthyretin (prealbumin, amyloidosis type I)**
25828	TXN2	thioredoxin 2
9097	USP14	ubiquitin specific peptidase 14 (tRNA-guanine transglycosylase)
27072	VPS41	vacuolar protein sorting 41 homolog (S. cerevisiae)
80344	WDR23	WD repeat domain 23
7507	XPA	xeroderma pigmentosum, complementation group A
7709	ZBTB17	zinc finger and BTB domain containing 17
10444	ZER1	zer-1 homolog (C. elegans)
7739	ZNF185	zinc finger protein 185 (LIM domain)

Housekeeping genes

**506**	**ATP5B**	**ATP synthase F1 subunit beta**
**7917**	**BAT3**	**BAG cochaperone 6**
**1351**	**COX8A**	**cytochrome c oxidase subunit 8A**
**3094**	**HINT1**	**histidine triad nucleotide binding protein 1**
**3181**	**HNRNPA2B1**	**heterogeneous nuclear ribonucleoprotein A2/B1**
**4695**	**NDUFA2**	**NADH:ubiquinone oxidoreductase subunit A2**

Supplemental table 1 (related to Figure 1): Prognostic liver signature (PLS) gene list. List of the 73 poor-prognosis genes and of the 113 good-prognosis genes of the PLS (13). The reduced version of the PLS corresponds to 32 genes bioinformatically selected and validated in patient cohort (10). These 32 genes are highlighted in blue. The 6 housekeeping genes used to normalize the PLS gene expression are also listed.

SUPPLEMENTAL TABLE 2

Huh7.5.1^{dif} + LX2

Phenotype	Gene set	NES	FDR q-val
HCV versus Mock	Poor-prognosis	1.66	0.04
HCV versus Mock	Good-prognosis	-1.33	0.176
Captopril versus HCV	Poor-prognosis	-1.4	0.148
Captopril versus HCV	Good-prognosis	0.92	0.54

Huh7.5.1^{dif} + LX2 + Macrophage

Phenotype	Gene set	NES	FDR q-val
FFA versus Mock	Poor-prognosis	2.7	0
FFA versus Mock	Good-prognosis	-1.87	0.014
Captopril versus FFA	Poor-prognosis	-1.8	0.023
Captopril versus FFA	Good-prognosis	0.82	0.743

Huh7.5.1^{dif}

Phenotype	Gene set	NES	FDR q-val
HCV versus Mock	Poor-prognosis	1.67	0.003
HCV versus Mock	Good-prognosis	-1.75	0.009
Losartan versus HCV	Poor-prognosis	-1.43	0.021
Losartan versus HCV	Good-prognosis	1.21	0.091

Angiotensin I

Phenotype	Gene set	NES	FDR q-val
AngI 1 µM versus Mock	Poor-prognosis	0.65	0.851
AngI 1 µM versus Mock	Good-prognosis	-1.4	0.148
AngI 10 µM versus Mock	Poor-prognosis	2.49	0.001
AngI 10 µM versus Mock	Good-prognosis	-1.47	0.078

Angiotensin II

Phenotype	Gene set	NES	FDR q-val
AngII 1 µM versus Mock	Poor-prognosis	0.83	0.719
AngII 1 µM versus Mock	Good-prognosis	-1.04	0.37
AngII 10 µM versus Mock	Poor-prognosis	1.25	0.235
AngII 10 µM versus Mock	Good-prognosis	-1.33	0.176

Supplemental table 2 (referring to figure 1): Normalized enrichment score (NES) and False discovery rates (FDR) of cell culture experiment for poor- and good-prognosis gene set of the PLS. Data were obtained using GSEA analysis with the following parameters (32 gene signature, Enrichment statistics = classic, Metric for ranking genes = tTest). For discovery in cell culture, the results are considered as significant if FDR < 0.25 according to GSEA (22). Validations were further performed in animal models.

SUPPLEMENTAL TABLE 3

Patient 1 (non-diseased-liver)

Phenotype	Gene set	NES	FDR q-val
FFA versus Mock	Poor-prognosis	1.66	0.026
FFA versus Mock	Good-prognosis	-1.21	0.229
Captopril versus FFA	Poor-prognosis	-1.44	0.087
Captopril versus FFA	Good-prognosis	1.68	0.022

Patient 2 (non-diseased-liver)

Phenotype	Gene set	NES	FDR q-val
FFA versus Mock	Poor-prognosis	3.06	0
FFA versus Mock	Good-prognosis	-2.75	0
Captopril versus FFA	Poor-prognosis	-2.39	0.001
Captopril versus FFA	Good-prognosis	2.25	0.002

Patient 3 (alcoholic liver disease)

Phenotype	Gene set	NES	p-value
Captopril versus DMSO	Poor-prognosis	-2.70	0.002
Captopril versus DMSO	Good-prognosis	0.70	0.198

Patient 4 (HBV)

Phenotype	Gene set	NES	p-value
Erlotinib versus DMSO	Poor-prognosis	-2.70	0.002
Erlotinib versus DMSO	Good-prognosis	2.23	0.006

Patient 5 (NASH)

Phenotype	Gene set	NES	p-value
Erlotinib versus DMSO	Poor-prognosis	-2.00	0.010
Erlotinib versus DMSO	Good-prognosis	2.05	0.009

Supplemental table 3 (referring to figure 7): Normalized enrichment score (NES) and False discovery rates (FDR) of spheroid experiment for poor- and good-prognosis gene set of the 186 PLS. Data were obtained using GSEA analysis (patients 1-2) or GSEI (patients 3 to 5).

SUPPLEMENTAL TABLE 4

Method	Patient	Internal number	Etiology	Fibrosis stage	Grade	Medical History	Treatments
Tumorspheroids (tumor tissues)	HCC1	394	No chronic liver disease	F0-1	G3	Diabetes, dyslipidemia	Metformin, sitagliptin, gliclazide
	HCC2	404	NASH	F1-2	G1	COPD, dyslipidemia, inactive Alchol Use Disorder, eradicated hepatitis C	Lamotrigin, escitalopram, amitriptylin, lorazepam, fenofibrate, tamsulosin, indacaterol/glycopirronium
	HCC3	419	HCV	F1	G2-3	Dyslipidemia, stroke	Allopurinol, acetylsalycilic acid, pantoprazole, pravastatin
	HCC4	489	NASH	F0	G3	Diabetes, hypertension, obesity (BMI 31), stroke, gastritis, diverticulosis, COPD, phlebitis	Apixaban, gliclazide, herbal extract
Spheroids (non-diseased tissues)	Patient 1	465	No chronic liver disease, neuroendocrine tumor	/	/	Nothing to report	No treatment
	Patient 2	456	CRLM	/	/	Nothing to report	No treatment
Precision cut liver slices	Patient 3	ev272	Alcohol	F3	/	Not available	Not available
	Patient 4	ev069	HBV	F1	/	Not available	Not available
	Patient 5	ev064	NASH	F3	/	Not available	Not available

Supplemental table 4 (related to Figure 7): Human liver tissues were obtained from patients undergoing liver resection with informed consent from all patients. Table summarizes patient characteristics. CRLM = colorectal liver metastases; HCV = hepatitis C virus; NASH = non-alcoholic liver disease. Spheroids were generated from liver tissue from patient without history of chronic liver disease. Tumorspheroids were generated from HCC tissues from HCC patients.

FULL UNEDITED GEL

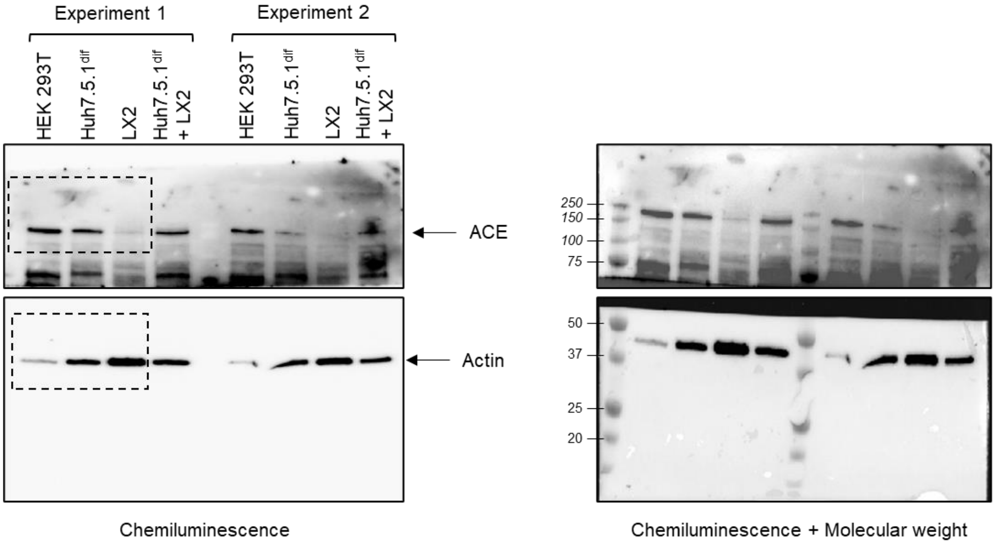

Full unedited gel for figure 1B. Full-length gels are shown for Figure 1B. Black dashed squares indicate the bands shown in the Figure 1B. Protein analysis was performed in cell lysates from two independent experiments using a reducing 12% SDS-PAGE gel electrophoresis. PVDF membrane was cut and probed for ACE and Actin. Proteins were visualized by chemiluminescence. The marker sizes (Precision Plus Protein Standards All Blue, BioRad) are indicated on the right panel (molecular weight, kDa). References of the antibodies are provided in Supplementary Material and Method.

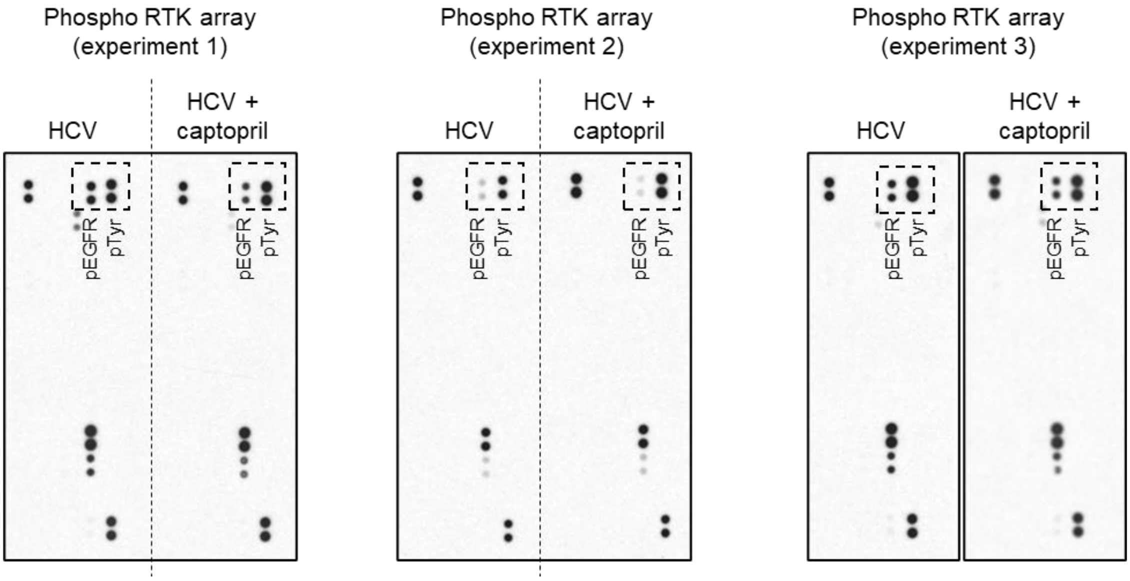

Full unedited array for figure 5B. Full-length arrays are shown for Figure 5B from Human Phospho-RTK Array kit (R&D Systems). Black dashed squares indicate the dots shown in the Figure 5B (EGFR phosphorylation + CTRL) and the dots used for the quantification of EGFR phosphorylation. Phosphorylation analysis was performed from cell lysates from three independent experiments according to manufacturer’s instructions.

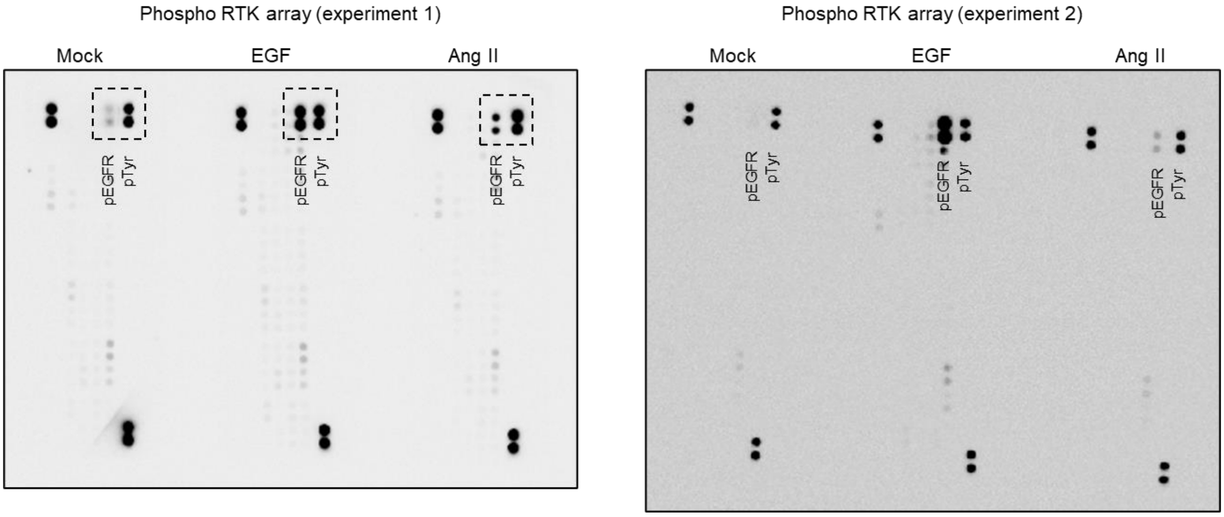

Full unedited array for figure 5C. Full-length arrays are shown for Figure 5C from Human Phospho-RTK Array kit (R&D Systems). Black dashed squares indicate the dots shown in the Figure 5C (EGFR phosphorylation + CTRL). Phosphorylation analysis was performed from cell lysates from two independent experiments according to manufacturer’s instructions.

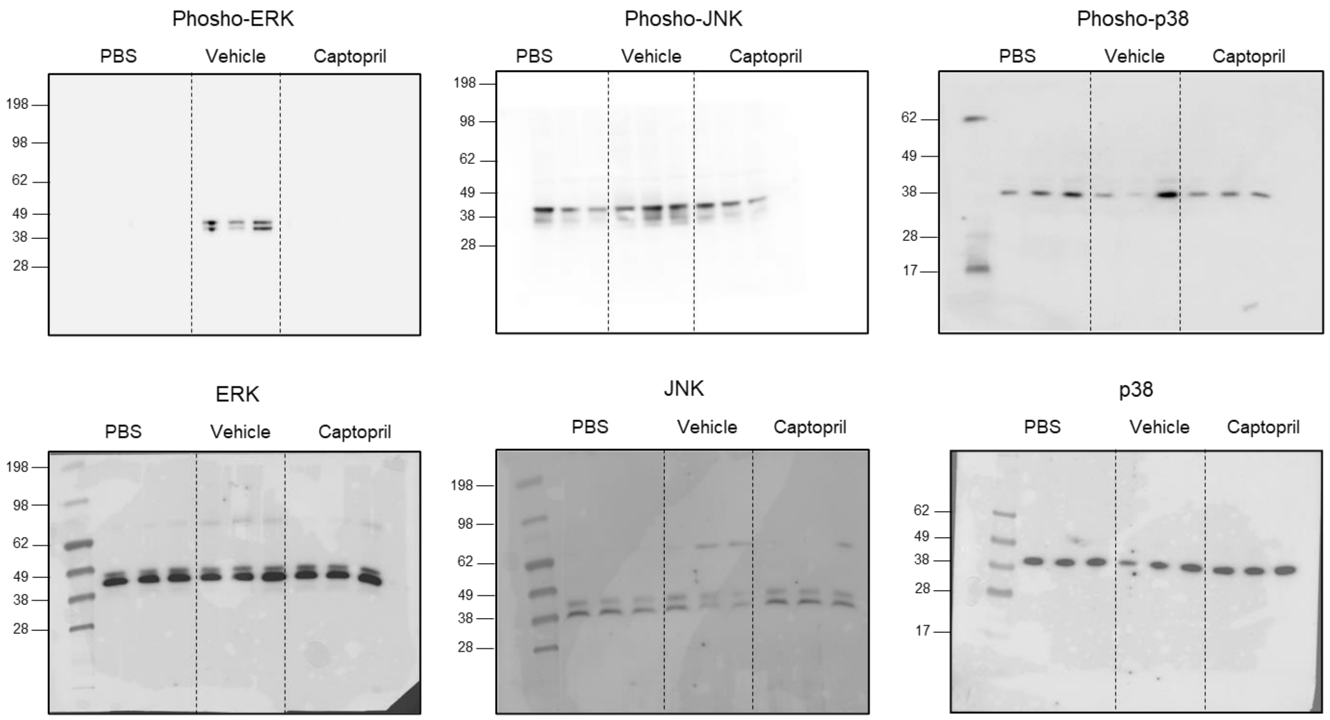

Full unedited gel for figure 5G. Full-length gels are shown for Figure 5G. Protein analysis was performed from mouse liver tissues (3 animal per groups) using a reducing 12% SDS-PAGE gel electrophoresis. Due to similar size of the proteins, analysis of total proteins and their phosphorylated forms was performed on different gels however using the same lysates from the same experiment. Proteins were visualized by chemiluminescence. The marker sizes (SeeBlue™ Plus2 Pre-stained Protein Standard, Life Technology) are indicated on each panel (molecular weight, kDa). References of the antibodies are provided in Supplementary Material and Method.

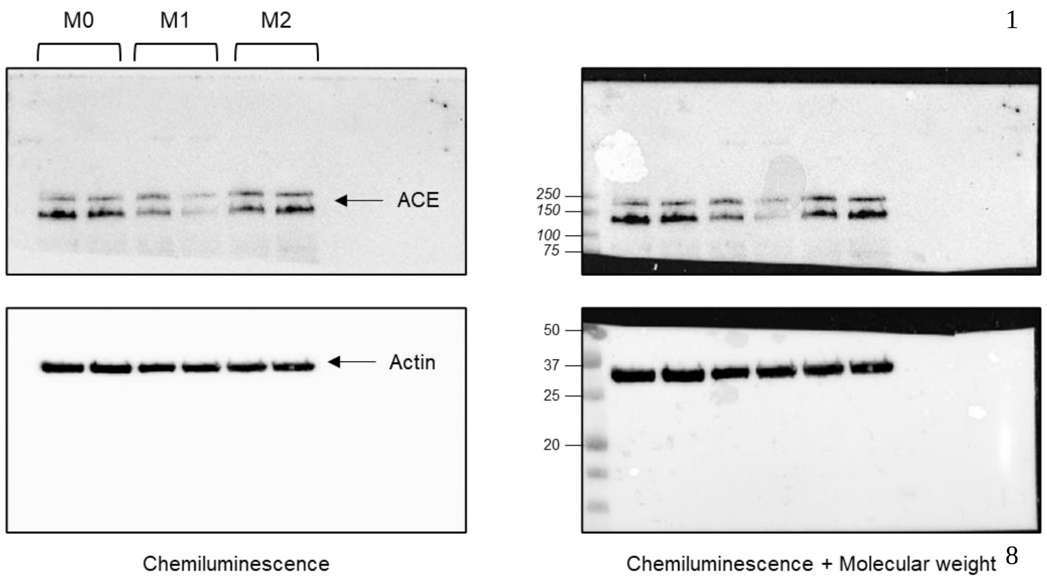

Full unedited gel for Supplementary figure 3C. Full-length gels are shown for Supplementary figure 3C. Protein analysis was performed in cell lysates from two independent experiments using a reducing 12% SDS-PAGE gel electrophoresis. PVDF membrane was cut and probed for ACE and Actin. Proteins were visualized by chemiluminescence. The marker sizes (Precision Plus Protein Standards All Blue, BioRad) are indicated on the right panel (molecular weight, kDa). References of the antibodies are provided in Supplementary Material and Method.

SUPLEMENTAL REFERENCES

1. Merz A et al. Biochemical and morphological properties of hepatitis C virus particles and determination of their lipidome. *J. Biol. Chem.* 2011;286(4):3018–3032.

2. Trombetta JJ et al. Preparation of Single-Cell RNA-Seq Libraries for Next Generation Sequencing. *Curr. Protoc. Mol. Biol.* 2014;107:4.22.1-17.

3. Shalek AK et al. Single-cell RNA-seq reveals dynamic paracrine control of cellular variation. *Nature* 2014;510(7505):363–369.

4. Xiao F et al. Hepatitis C Virus Cell-Cell Transmission and Resistance to Direct-Acting Antiviral Agents. *PLOS Pathog.* 2014;10(5):e1004128.

5. Trapnell C et al. Differential gene and transcript expression analysis of RNA-seq experiments with TopHat and Cufflinks. *Nat. Protoc.* 2012;7(3):562–578.

6. Kim D et al. TopHat2: accurate alignment of transcriptomes in the presence of insertions, deletions and gene fusions. *Genome Biol.* 2013;14:R36.

7. Roberts A, Pimentel H, Trapnell C, Pachter L. Identification of novel transcripts in annotated genomes using RNA-Seq. *Bioinforma. Oxf. Engl.* 2011;27(17):2325–2329.

8. Peck D et al. A method for high-throughput gene expression signature analysis. *Genome Biol.* 2006;7:R61.

FULL UNEDITED GEL

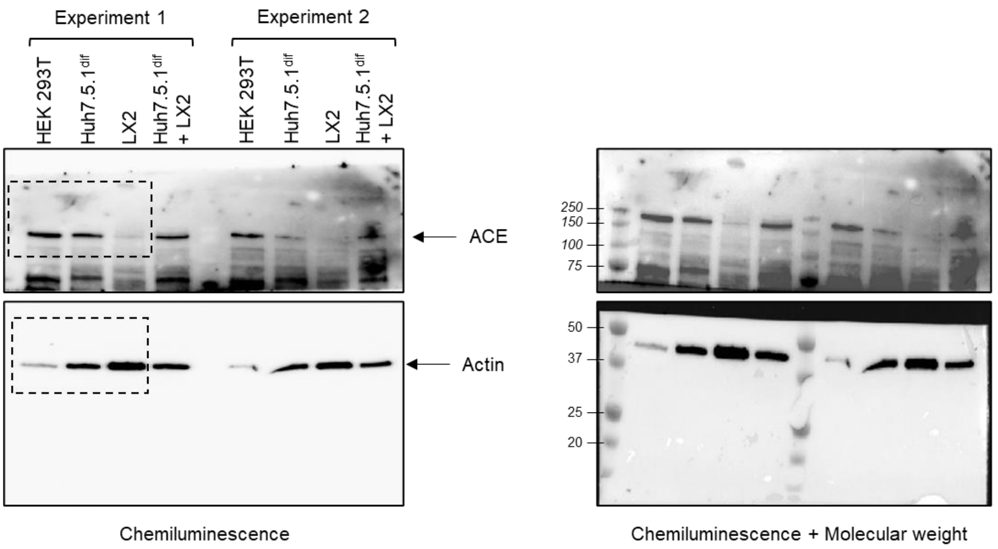

Full unedited gel for figure 1B. Full-length gels are shown for Figure 1B. Black dashed squares indicate the bands shown in the Figure 1B. Protein analysis was performed in cell lysates from two independent experiments using a reducing 12% SDS-PAGE gel electrophoresis. PVDF membrane was cut and probed for ACE and Actin. Proteins were visualized by chemiluminescence. The marker sizes (Precision Plus Protein Standards All Blue, BioRad) are indicated on the right panel (molecular weight, kDa). References of the antibodies are provided in Supplementary Material and Method.

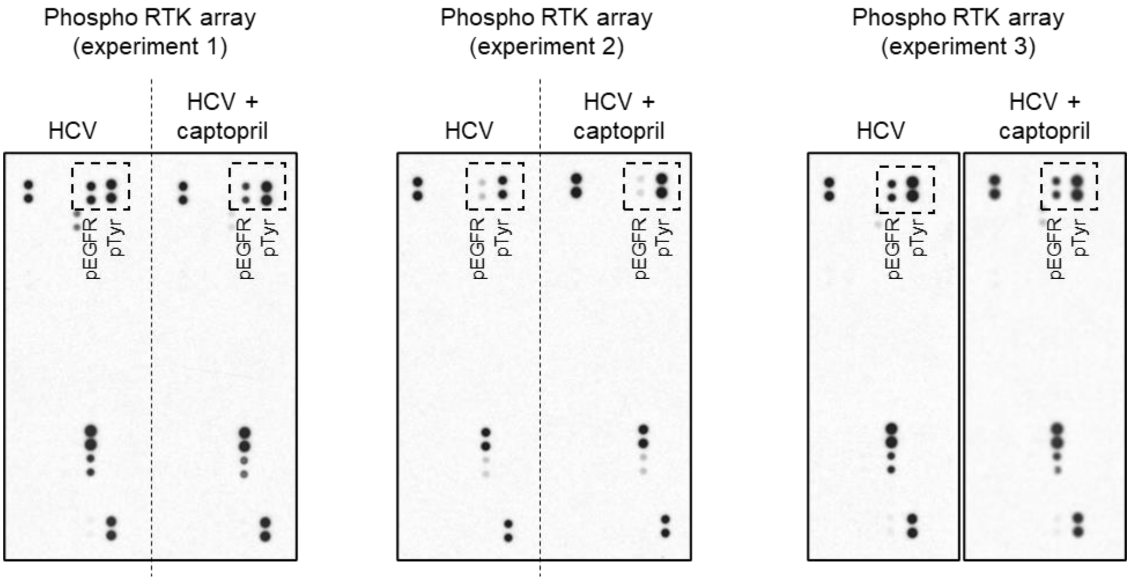

Full unedited array for figure 5B. Full-length arrays are shown for Figure 5B from Human Phospho-RTK Array kit (R&D Systems). Black dashed squares indicate the dots shown in the Figure 5B (EGFR phosphorylation + CTRL) and the dots used for the quantification of EGFR phosphorylation. Phosphorylation analysis was performed from cell lysates from three independent experiments according to manufacturer's instructions.

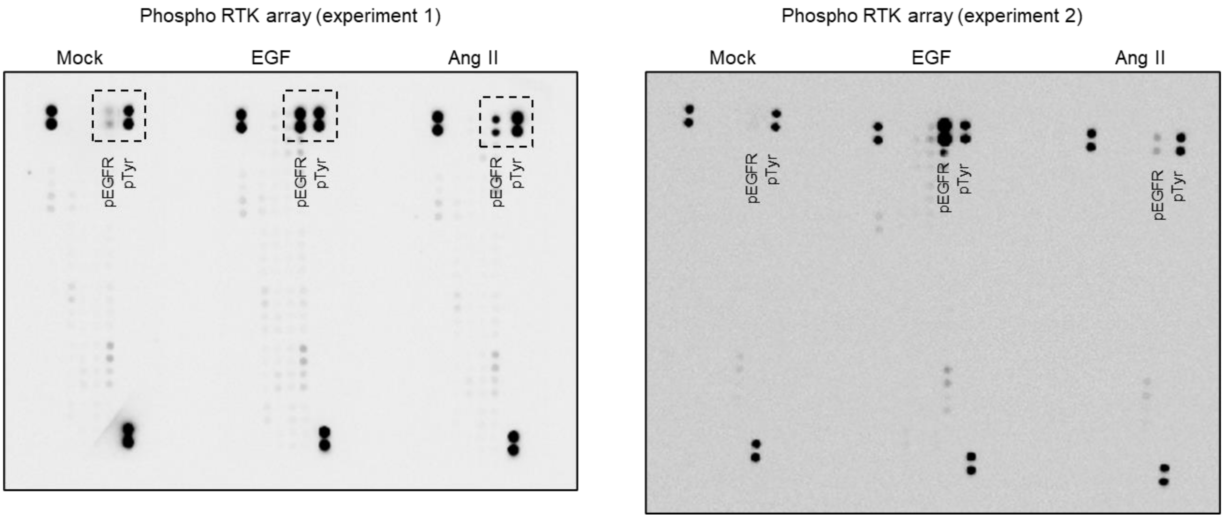

Full unedited array for figure 5C. Full-length arrays are shown for Figure 5C from Human Phospho-RTK Array kit (R&D Systems). Black dashed squares indicate the dots shown in the Figure 5C (EGFR phosphorylation + CTRL). Phosphorylation analysis was performed from cell lysates from two independent experiments according to manufacturer's instructions.

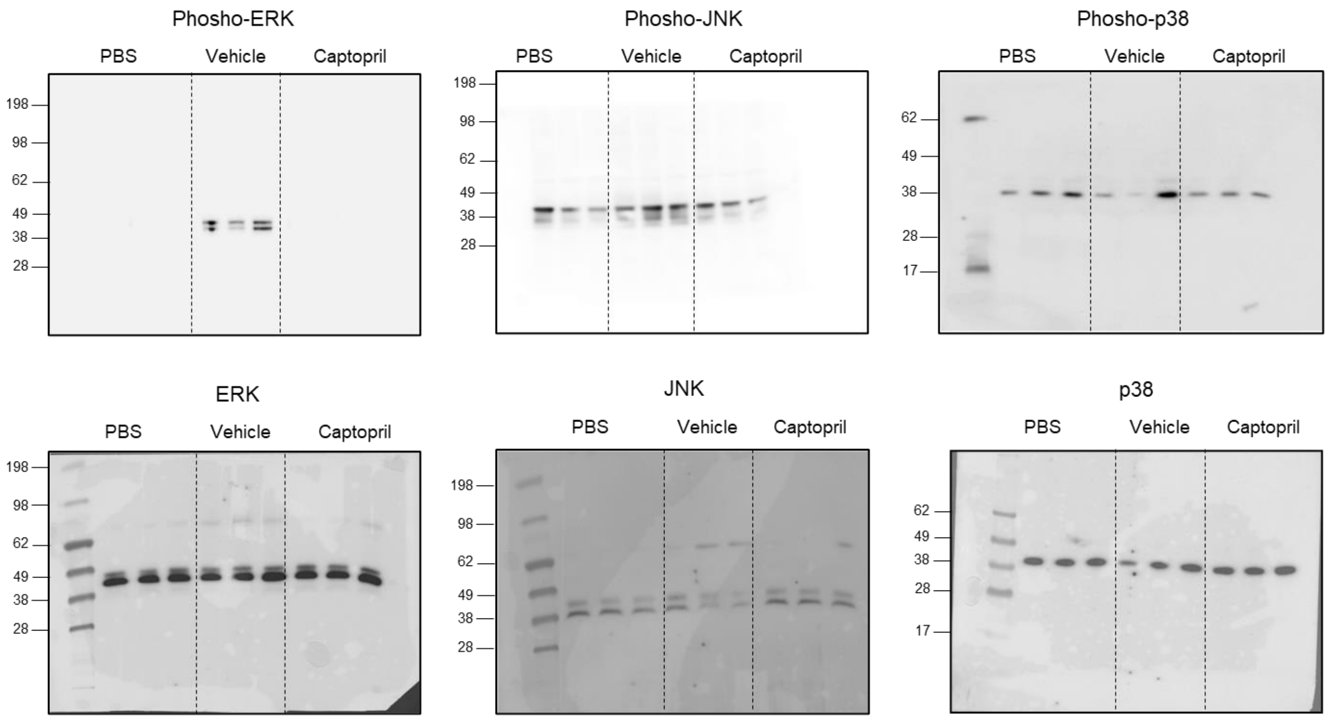

Full unedited gel for figure 5G. Full-length gels are shown for Figure 5G. Protein analysis was performed from mouse liver tissues (3 animal per groups) using a reducing 12% SDS-PAGE gel electrophoresis. Due to similar size of the proteins, analysis of total proteins and their phosphorylated forms was performed on different gels however using the same lysates from the same experiment. Proteins were visualized by chemiluminescence. The marker sizes (SeeBlue™ Plus2 Pre-stained Protein Standard, Life Technology) are indicated on each panel (molecular weight, kDa). References of the antibodies are provided in Supplementary Material and Method.

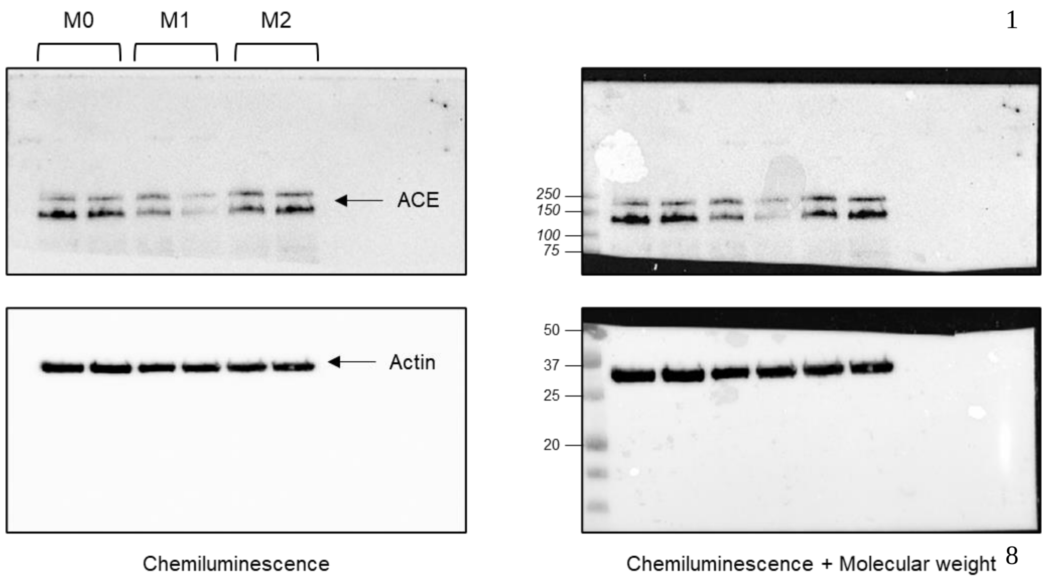

Full unedited gel for Supplementary figure 3C. Full-length gels are shown for Supplementary figure 3C. Protein analysis was performed in cell lysates from two independent experiments using a reducing 12% SDS-PAGE gel electrophoresis. PVDF membrane was cut and probed for ACE and Actin. Proteins were visualized by chemiluminescence. The marker sizes (Precision Plus Protein Standards All Blue, BioRad) are indicated on the right panel (molecular weight, kDa). References of the antibodies are provided in Supplementary Material and Method.
